# Spatially context aware multilevel color image segmentation using a Hybrid Artificial Hummingbird and Great Deluge mechanism

**DOI:** 10.1038/s41598-026-56525-2

**Published:** 2026-06-12

**Authors:** Tirumalasetti Supraja, Kankanala Srinivas

**Affiliations:** School of Electronics Engineering, VIT-AP University, Inavolu, Beside AP Secretariat, Amaravati, 522237 Andhra Pradesh India

**Keywords:** Artificial Hummingbird Algorithm, Minimum cross entropy, Image segmentation, Multilevel thresholding, Energy curve, Great Deluge Algorithm, Engineering, Mathematics and computing

## Abstract

Multilevel image thresholding is an important segmentation technique that partitions an image into meaningful regions in applications such as object recognition, medical imaging, and satellite image analysis. However, conventional techniques are limited by their poor sensitivity to initial conditions, lack of sufficient spatial contextual information, and slow convergence. The proposed method uses a novel hybrid model based on the Artificial Hummingbird Algorithm (AHA) to address the limitations of existing approaches. Here, Latin Hypercube, Sobol, Halton, and Sierpinski strategies are used during the population initialization phase to improve population diversity and search space coverage. This improves the exploration capability of the algorithm and supports better search space. The proposed methodology also uses spatial contextual information to improve the quality of segmentation. It also incorporates a relationship between neighboring pixels in order to retain more structure and enhance the visual performance of the output. For the exploitation phase, the Great Deluge Algorithm (GDA) is utilized as the optimization algorithm. Furthermore, the use of GDA serves as an adaptive acceptance function which reduces the chance of getting stuck during the search process. Minimum Cross Entropy Measure (MCEM) is used as the objective function to obtain optimal threshold values. Different evaluation metrics have been used to compare the results of the proposed method with other existing metaheuristic algorithms. The code is available at https://github.com/suprajatirumalasetti/AHA_GDA_Image_Segmentation_Code.

## Introduction

The process of image segmentation has a major role to play in the field of computer vision and image processing, since it helps in partitioning the image into several meaningful components for further processing. The process of image segmentation holds significance for the analysis and interpretation of various kinds of images such as medical images, satellite images, natural images, and so forth. This method has been used in medical imaging as well as industrial inspection^1^. The optimization of threshold values is an NP-hard problem; therefore, exhaustive search becomes computationally impractical. In this context, bio inspired metaheuristic algorithms are useful for handling nonlinear and multimodal search spaces^2^.

The foraging behavior of hummingbirds has recently been modeled in Artificial Hummingbird Algorithm (AHA) proposed by Zhao et al.^3^. AHA balances local exploitation and global exploration through guided foraging, territorial foraging, migration foraging, and a memory mechanism that records visited food sources. Despite its effectiveness, AHA could be subject to some weaknesses common to population based approaches, such as early convergence, dependency on the initial conditions, and local optimum. Two critical techniques that can enhance AHA include improvement in the criterion of acceptance for the candidate solution and better population coverage through initialization.

The AHA has been combined with Great Deluge Algorithm (GDA)^4^ that utilizes the water level concept for accepting candidate solution. By using this, the system can accept non improving solutions with control due to change in water level, thus making the system efficient in overcoming local minima. Like Simulated Annealing (SA), GDA has been proved to work well in finding near optimal solution in large scale optimization problem. Also, like randomization, random initialization can cause candidates to be initialized at limited regions of the search space, resulting in less diversity in population as well as search space. Therefore, four different types of initialization scheme have been employed in this proposed method: Latin Hypercube Sampling (LHS)^5^, Sobol, Halton, and Sierpinski initialization^6^. The performance of multilevel thresholding largely depends upon the fitness function chosen. Entropy criteria is popularly used in segmentation and Minimum Cross Entropy Measure (MCEM) works well since it minimizes the distributional distance between the two distributions^7^. Unlike variance based OTSU^8^ criterion, MCEM relies on distributional criteria which make it more suitable for multilevel thresholding.

In contrast to conventional histograms that are mainly focused on pixel intensities, this paper presents an approach using energy curves to assist in finding the best possible thresholds^9,10^. An energy curve can help to smooth the histogram and account for spatial context by offering a continuous version of the histogram, leading to better results concerning the detection of the threshold region and therefore avoiding structure loss, maintaining segmentations borders, and providing a better balance of exploration and exploitation. We offer a mixed AHA approach with MCEM used as a fitness function, GDA as the accepting criteria and four initialization approaches. Our framework is tested using two benchmark datasets: BSDS500 for natural images^11^ and NASA Earth Observatory dataset (NASA) for satellite images^12^.

The key contributions of our approach are:

- Energy curve is obtained by means of smoothing of histograms and taking into account spatial context; the continuous landscape obtained defines the threshold locations, retains structural information, and stabilizes the segmentation process
- The proposed method combines AHA and GDA methods for segmentation using thresholding at multiple levels; this combination increases exploration capability and avoids early convergence.
- There are four strategies for initialization of algorithm parameters, namely Latin Hypercube sampling, Sobol, Halton, and Sierpinski used to improve solution diversity and optimization performance.
- The proposed framework is validated on the BSDS500 dataset for natural images and the NASA Earth Observatory dataset for satellite images, with MCEM used as the objective function.
- Extensive experiments using Peak Signal to Noise Ratio (PSNR)^13^, Structural Similarity Index Measure (SSIM)^14^, Feature Similarity Index Measure (FSIM)^15^, Quality Index based on Local Variance (QILV)^16^, Correlation Coefficient (CORR)^17^, Edge Preservation Index (EPI)^18^, and Mutual Information Factor (MIF)^19^, along with statistical significance tests, show that the proposed method provides reliable and competitive performance compared with recent metaheuristic algorithms.

The rest of this paper is organized as follows. Section “Literature review” reviews the relevant literature on multilevel thresholding techniques, optimization based segmentation and metaheuristic algorithms. Section “Proposed work” describes the proposed algorithm for multilevel thresholding, including a pixel wise energy curve to catch local intensity variations. This gives more details to the integration of AHA with MCEM, population initialization strategies for enhanced diversity and local refinement via GDA. Section “Results and discussion” presents the experimental results and corresponding discussion, while section “Conclusion” concludes the paper and highlights directions for future research.

## Literature review

Image segmentation is the most vital preprocessing stage in image processing where an image is divided into significant parts also known as image thresholding. Over the past few decades, numerous thresholding techniques have been introduced in the literature^1^. Threshold segmentation is the most commonly used method for image segmentation^20^. Accurate and robust segmentation requires consistent pixel attributes throughout each region^21^. Threshold based segmentation approaches in image segmentation are often classified into two types^22^. Bilevel thresholding is a fundamental technique that segments an image into two separate classes by determining a single optimal threshold value, it is insufficient for satellite image segmentation^23^. On the other hand, multilevel thresholding partitions the image into multiple segments by locating several threshold values.

Multilevel thresholding is a straightforward and efficient technique in image classification nevertheless, the cost of computation is expensive as the levels of threshold rise. Satellite images have a variety of distinct and complicated objects: the territory, plants, buildings, mountain, urban regions, etc. These areas are rather difficult to differentiate in the poor quality of the image and illumination. Bilevel thresholding is thus inadequate where a satellite image segmentation^23^. Over the past few decades, literature has reported over hundreds of ways in which the global thresholding can be performed as a part of image segmentation processes^24^. The techniques of thresholding are classified as parametric and nonparametric models. Nonparametric models seek the thresholds, which maximize an objective function, introduced by Otsu^8^ and Kapur^25^. In 1988, Sahoo concluded that the best thresholding image segmentation method was as illustrated by Otsu and gained popularity in literature^24,26,27^. Nonetheless, the intensive search conditions of the Otsu algorithm are relatively time consuming, and thus have been replaced by various metaheuristic (optimization) algorithms to overcome this problem. Inspired by the natural life systems, the predominant metaheuristic algorithms include the Genetic Algorithm (GA)^28^, Differential Evolution (DE)^29^, the Artificial Bee Colony (ABC)^30^, Particle Swarm Optimization (PSO)^31^, and the Moth Flame Optimization (MFO)^32^, Aquila Optimization (AO)^33^, Equilibrium Optimization (EO)^34^, and Whale Optimization Algorithm (WOA)^35^.

The no free lunch theorem (NFLT) states that in every problem no single optimization algorithm can be superior to any other algorithm^36^. From the above survey, classical histogram based methods like the Otsu^8^ and Kapur often fail to capture spatial contextual information, leading to inconsistent segmentation performance in multilevel thresholding. Recent studies have proposed energy curve model that averages the histogram and reflects the information of the neighboring areas of the image and thus provides a more accurate representation of the image structures^37,38^. Even if the thresholding algorithm using the metaheuristic is useful in simplifying images, retaining spatial context information becomes difficult. The theoretical basis for doing so is possible through spatial processing methods, which include the mathematics of image processing based on diffusions^39^, as well as edge preservation of structures used in focusing in multiple images^40^. Taking cues from such spatial preservation concepts, the energy curve model suggested in our paper takes into consideration the interaction among neighboring pixels.

Energy curve driven segmentation approaches were explored in^37,38^, and employing Electro Magnetism Optimization (EMO) and the Smell Agent Optimizer to enhance multilevel threshold selection and segmentation performance^41^. Although there are significant improvements in the multilevel thresholding and metaheuristic based image segmentation, none of the studies has been done to synthesize structured initialization strategies and energy curve modeling particularly when it involves RGB image segmentation. Furthermore, the two factors have not been combined into a hybrid architecture where the AHA and the GDA acceptance mechanism. Although Hybrid framework based on energy curves from AHA^42^ and Hybrid CPA^43^ have significantly enhanced the capability of multilevel color image segmentation; however they still do not include the integration of GDA, and the use of different heterogeneity initialization strategies.

To address this gap, this paper proposes a new standard of segmentation that (i) employs four structured patterns of initializations (Latin Hypercube, Sobol, Halton and Sierpinski) to enhance the diversity and stability of the population (ii) uses energy curve modelling to increase the contextual fidelity of threshold values and (iii) extends the process to RGB images, thereby guaranteeing a strong and stable performance. The behavior of the suggested approach was confirmed by comparing it with the rest of the state of the art metaheuristics such as the AO, EO, PSO, WOA, and the original AHA algorithms.

Table 1 presents a qualitative summary of the related literature by comparing existing thresholding and metaheuristic based segmentation methods in terms of key methodologies, objective criteria, qualitative metrics considered, strengths, limitations, and research gaps. This comparison highlights the limitations of conventional histogram based methods, standard metaheuristic algorithms, and initialization strategies, convergence stability, and population diversity.

**Table 1:** Qualitative summary of related literature and identified research gaps

Category	Key methodologies	Objective / criterion	Qualitative metrics considered	Strengths, limitations, and research gaps
Traditional thresholding	Otsu^8^, Kapur^25^	Variance based and entropy based thresholding	Histogram distribution and gray level intensity information	These methods are simple and widely used for basic thresholding tasks. However, they mainly depend on histogram information and do not include spatial contextual information, which may reduce stability in complex multilevel segmentation
Standard metaheuristics	GA^28^, DE^29^, ABC^30^, PSO^31^, MFO^32^, AO^33^, EO^34^, WOA^35^	Optimization of threshold values using entropy or threshold-based fitness functions	Exploration ability, exploitation behaviour, convergence tendency, and threshold search performance	These methods reduce the computational burden of exhaustive threshold search. However, they may suffer from premature convergence and local optima stagnation in high dimensional search spaces, as suggested by the No Free Lunch theorem
Initialization strategies	Latin Hypercube^5^, Sobol, Halton, Sierpinski^6^	Population diversity and search space coverage	Initial solution distribution, population spread, and exploration stability	Structured initialization improves initial population diversity and reduces clustering of candidate solutions. Poorly structured initialization can lead to inefficient exploration and unstable convergence behaviour

## Proposed work

The proposed framework combines structured population initialization, energy curve based threshold evaluation, AHA search behaviour, and GDA based acceptance refinement for RGB multilevel image segmentation. The detailed mathematical steps are explained in the following subsections.

### Energy curve based multilevel thresholding

**Fig. 1:**
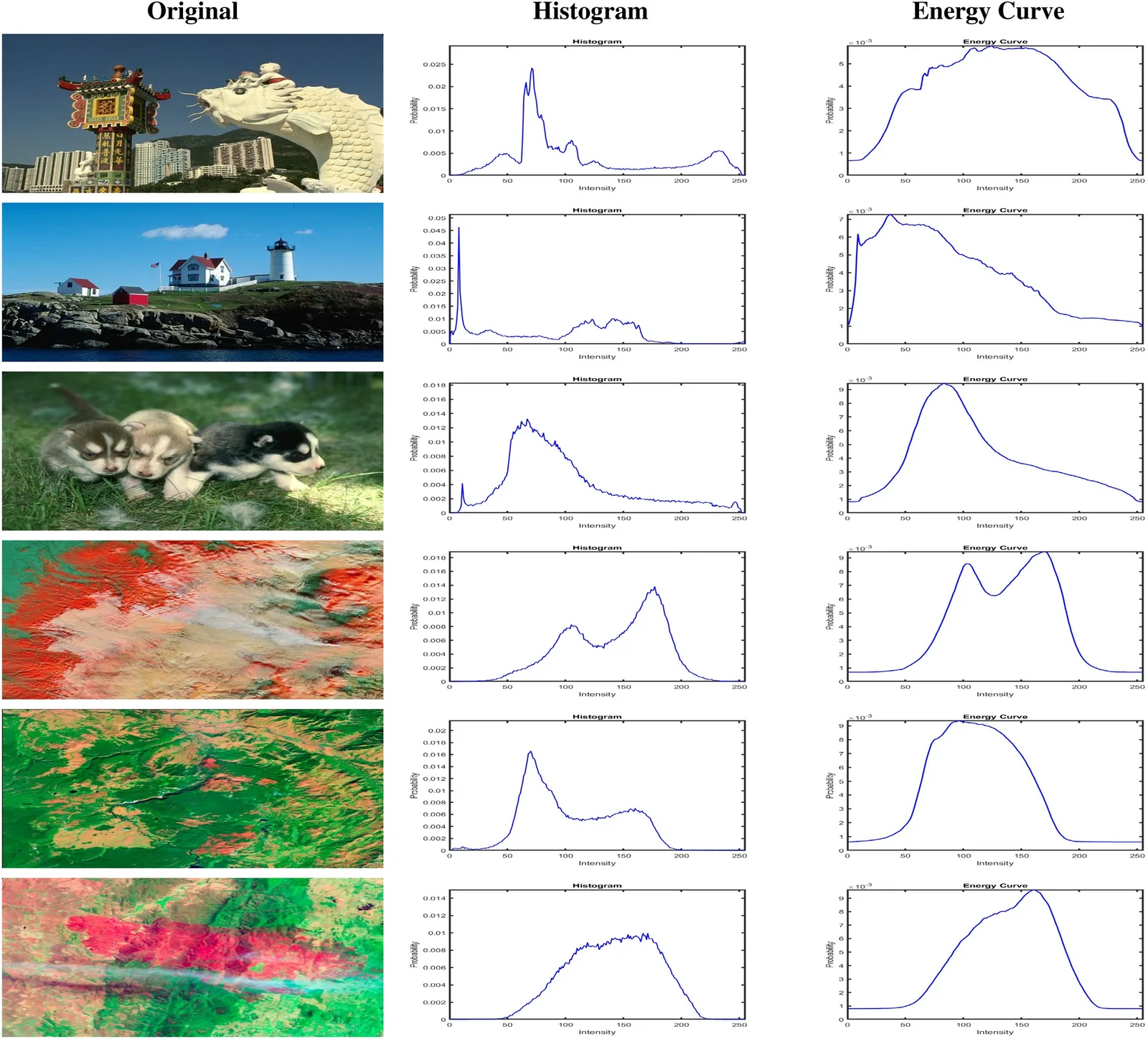
Comparison of original images, histograms, and energy curves for six test samples from the BSDS500 and NASA datasets.

Conventional histogram based methods do not effectively incorporate spatial contextual information when selecting suitable threshold values. The proposed method introduces an energy curve based multilevel thresholding strategy to address these limitations. The energy curve of an image is obtained via the modified Hopfield neural network^9,10^. The Hopfield neural network comprises several neurons, each interconnected by symmetric synaptic weights that exclude any self feedback loops. Hopfield formulated the energy function, which encapsulates the network’s total state by considering its entire architecture. In the Hopfield neural network, each pixel of the image is treated as a neuron, and its energy is computed. In addition to using pixel intensity distribution, it also uses adjacent interactions to capture local spatial patterns. The proposed method is applied to color images by performing multilevel segmentation on each RGB channel separately, in contrast to grayscale thresholding algorithms. A comparison of the original images, their corresponding histograms, and energy curves for six test samples is illustrated in Fig. 1.

**Algorithm 1:**
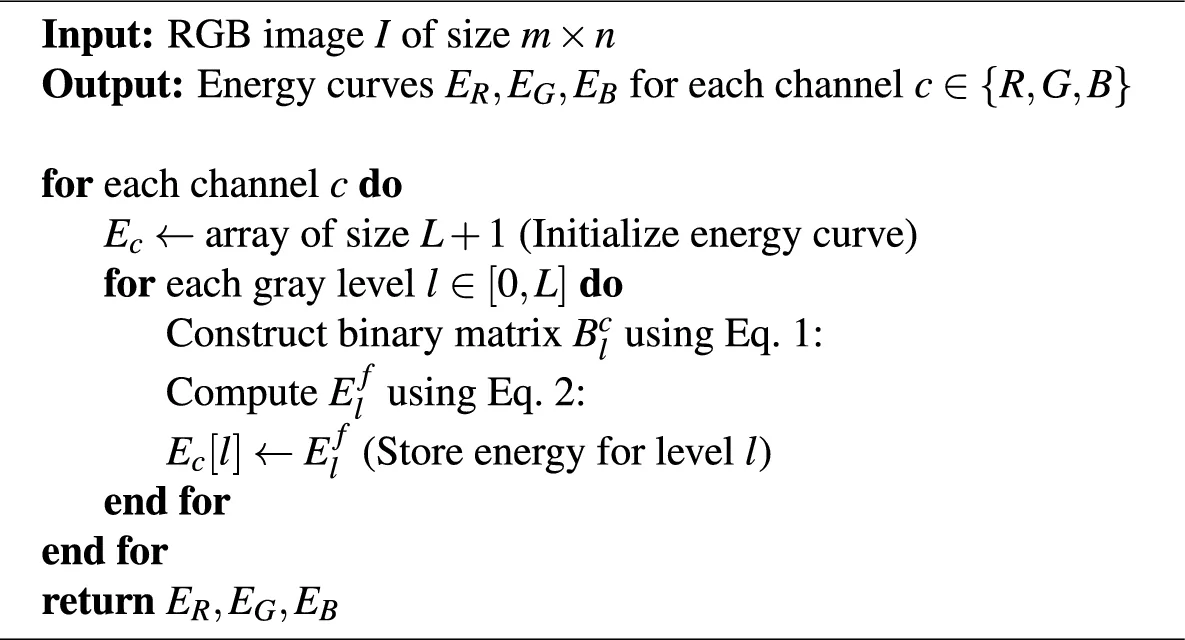
Energy curve computation.

Let the input images *I* be a color image composed of three channels $I=\{{{I}_{R}},{{I}_{G}},{{I}_{B}}\}$, where each channel ${{I}^{c}}$ (for $c\in \{R,G,B\}$ is treated as a grayscale image of size *m× n*. For each channel, let $g_{ij}^{c}$ represents at pixel location (*i*, *j*) . For a given spatial position (*i*, *j*), the neighborhood system *N* of order *X* is defined as follows: $N_{ij}^{X}=(i+u,j+v)$, where $(u,v)\in {{N}^{X}}$^10^. This defines the spatial correlation between nearby pixels of the image *I*. The configuration of the neighborhood system *N* varies based on order *X*^9^. $(u,v)\in \{(\pm 1,0),(0,\pm 1),(1,\pm 1),(-1,\pm 1)\}$ is that of the second order neighborhood system $N_{ij}^{X}$. In Table 2, the pixel at (*i*, *j*) spatial location is represented by its second order neighbor pixels. This 8 connectivity system enables modeling of local pixel interaction for energy evaluation. The neighborhood size acts as a tunable parameter dictating the algorithm’s sensitivity to local intensity shifts. Increasing the neighbourhood window can capture wider spatial information, but it also increases the computational cost. Very large windows are not suitable for precise thresholding because they may over-smooth the energy curve and blur fine regional boundaries. A tightly localized neighborhood is widely adopted in current segmentation literature because it strikes the necessary balance, preserving structural details while maintaining computational efficiency. Algorithm 1 summarizes the energy curve computation for RGB images.

**Table 2:** ${{N}^{2}}$ neighborhood of pixel (*i*, *j*).

$(i\!-\!1,\ j\!-\!1)$	$(i\!-\!1,\ j)$	$(i\!-\!1,\ j\!+\!1)$
$(i,\ j\!-\!1)$	*(i,\ j)*	$(i,\ j\!+\!1)$
$(i\!+\!1,\ j\!-\!1)$	$(i\!+\!1,\ j)$	$(i\!+\!1,\ j\!+\!1)$

For each gray level *l∈ [0,L]* , *L* is the maximum gray value i.e. 255, a binary matrix $B_{l}^{c}=\{b_{ij}^{c}\}$ is constructed in Eq. (1) for channel c such that:

1 $$\begin{aligned} b_{ij}^{c} = {\left\{ \begin{array}{ll} 1, & \text {if } b_{ij}^{c} \le l \\ -1, & \text {otherwise} \end{array}\right. } \end{aligned}$$

In the binary matrix $B_{l}$, every element $b_{ij}$ is assigned a value of either 1 or *-1*, based on $b_{ij}$ of the corresponding pixel and the value of *b*. Consider a constant matrix $Z = \{z_{ij} \mid 1 \le i \le m,\, 1 \le j \le n\}$ such that $z_{ij} = 1$ for all spatial positions (*i*, *j*). For the image *I*, the energy level *l* is computed as

2 $$\begin{aligned} {{E}^{f}}_{l}=-\sum \limits _{i=1}^{m}{\sum \limits _{j=1}^{n}{\sum \limits _{N_{ij}^{2}}{{{b}_{ij}}}}}{{b}_{pq}}+\sum \limits _{i=1}^{m}{\sum \limits _{j=1}^{n}{\sum \limits _{N_{ij}^{2}}{{{z}_{ij}}}}}{{z}_{pq}} \end{aligned}$$

where (*p*, *q*) are coordinates of neighboring pixels of the pixel at (*i*, *j*), *f=1* for grayscale image and $f=\{1,2,3\}$ for a color image. To transition from the single-level energy evaluation in Eq. (3) to the complete 1D energy curve used for thresholding, the total spatial energy ${E^{f}}_{l}$ must be calculated for every possible gray level *l ∈ [0, L]*. In this way, the distinct energy states are grouped together to form a single continuous 1*D* landscape that can be described mathematically using the vector:

3 $$\begin{aligned} \textbf{E}^{f} = [ {E^{f}}_{0}, {E^{f}}_{1}, \dots , {E^{f}}_{L} ] \end{aligned}$$

Where the components are the spatial energy states of the entire image, taking into account the current threshold. Using this single dimension array, the vector $\textbf{E}^{f}$ replaces the typical one-dimensional intensity histogram, which is spatial in nature.

### Minimum cross entropy for multilevel thresholding

In multilevel thresholding, the objective is to find threshold values that divide the image into meaningful regions. In the proposed method, MCEM is used as the objective function because it minimizes the difference between the original and segmented images. The related mathematical formulation is given below.

Cross entropy was first presented by Kullback^44^ in 1968 under the name of directed divergence. Information theory is measured using cross entropy. The cross entropy between the two probability distributions $A=\{{{A}_{1}}, {{A}_{2}}, ...... {{A}_{n}}\} \&\text {B}= \{{{\text {B}}_{1}}, {{\text {B}}_{2}}, ...... {{\text {B}}_{n}}\}$ is defined in Eq. (4):

4 $$\begin{aligned} c(A,B)=\sum \limits _{x=0}^{l-1}{{{A}_{x}}\log \frac{{{A}_{x}}}{{{B}_{x}}}} \end{aligned}$$

Where ${{A}_{x}}$ and ${{B}_{x}}$ denote the pixel locations in the reference image and the segmented image, respectively.

This can be regarded as an adaption of the maximum entropy method, wherein all ${{B}_{x}}$ are originally allocated identical estimates. MCEM thresholding identifies the optimal threshold value by minimizing the cross entropy between the original image and its thresholded version. Let *h*(*x*) represent the histogram associated with the intensity levels *x = 1, 2, … , l-1*, where *l* denotes the total number of gray levels, and let *I* signify the original image. The thresholded image $I_{TH}$ was calculated using the threshold value (TH). The class wise mean intensity, as specified in Eq. (6), is calculated over the relevant histogram interval and is utilized in the formulation of reference probability distributions for MCEM.

5 $$\begin{aligned} I_{TH}(x,y) = {\left\{ \begin{array}{ll} \mu (1, TH), & \text {if } I(x,y) < TH \\ \mu (TH, l+1), & \text {if } I(x,y) \ge TH \end{array}\right. } \end{aligned}$$

Where,

6 $$\begin{aligned} \mu (u,v)=\sum \limits _{x=u}^{v-1}{xh(x)/}\sum \limits _{x=u}^{v-1}{h(x)} \end{aligned}$$

The cross entropy is computed by reformulating Eq. (4) as shown below to derive the entropy value, which serves as the objective function (commonly referred to as the fitness function)^44^:

7 $$\begin{aligned} \begin{aligned} F_{\text {cross}}(TH) = \sum \limits _{x=0}^{TH-1} x h(x) \log \left( \frac{x}{\mu (1, TH)} \right) \\ + \sum \limits _{x=TH}^{l-1} x h(x) \log \left( \frac{x}{\mu (TH, l-1)} \right) \end{aligned} \end{aligned}$$

With the extension of Eq. (7) to a multilevel method, this objective function takes into account a threshold value for bilevel thresholding^41^. Subsequently, Eq. (7) is formulated, and its explicit expression is given in Eq. (8)^44^.

8 $$\begin{aligned} \begin{aligned} F_{\text {cross}}(TH) = \sum \limits _{x=0}^{l-1} x h(x) \log (x) \\ - \sum \limits _{x=0}^{TH-1} x h(x) \log \left( \mu (1, TH) \right) \\ - \sum \limits _{x=TH}^{l-1} x h(x) \log \left( \mu (TH, l-1) \right) \end{aligned} \end{aligned}$$

Based on the vector $TH=[T{{H}_{1}},T{{H}_{2}},....T{{H}_{tt}}]$, which comprises tt distinct threshold values, Eq. (7) multilevel method is carried out through the next Eqs. (9) and (10):

9 $$\begin{aligned} F_{\text {cross}}(\textbf{TH}) = \sum _{x=0}^{l-1} x h(x) \log (x) - \sum _{i=1}^{t} h_i \end{aligned}$$

where $h_i$ is defined as follows and tt is the total number of thresholds.

10 $$\begin{aligned} \begin{aligned} h_1&= \sum _{x=1}^{TH_1 - 1} x h(x) \log \left( \mu (1, TH_1) \right) \\ h_k&= \sum _{x=TH_k}^{TH_{k+1} - 1} x h(x) \log \left( \mu (TH_k, TH_{k+1}) \right) , \quad 1< k < tt \\ h_{tt}&= \sum _{x=TH_{tt}}^{l - 1} x h(x) \log \left( \mu (TH_{tt}, l - 1) \right) \end{aligned} \end{aligned}$$

### Population initialization strategies for enhanced diversity

Initialization of the population plays an important role in the convergence stability and exploration ability of metaheuristic algorithms. In the proposed method, Latin Hypercube, Halton, Sobol, and Sierpinski strategies are jointly used during initialization. The combined effect of this initialization technique helps improve the diversity of the population, along with preventing premature convergence of the population in the search space. The exact mathematics involved in these initialization techniques is described in the following sub-sections. The initial population is divided into four parts, where each part is generated using one of these techniques.

#### Latin hypercube

Mathematically, the *K*-th sample point for the *i*-th dimension, denoted as $x_{i}^{K}$, is generated using the following formulation:

11 $$\begin{aligned} x_{i}^{K} = \frac{\pi _{i}(K) - 1 + U_{i}^{K}}{N} \end{aligned}$$

where *N* represents the total number of partitions (which corresponds to the sub-population size), and $K \in \{1, 2, \dots , N\}$. The term $\pi _{i}$ denotes a random permutation function applied to the sequence of integers $\{1, 2, \dots , N\}$ for the *i*-th dimension. This permutation ensures that the samples do not align diagonally but are stochastically scattered. Finally, $U_{i}^{K}$ represents an independently and uniformly distributed random variable sampled within the continuous interval [0, 1]. By incorporating the permutation function and the uniform random variable, LHS prevents premature spatial clustering and ensures comprehensive global exploration during the initial iterations.

#### Sobol

Sobol sequence is designed with the aim of minimizing discrepancy using bitwise operations thus giving well spaced points in high dimensional space, is represented by using Eq. (12)^5^.

12 $$\begin{aligned} S(i)=\underset{j=1}{\overset{M}{\mathop {\oplus }}}\,{{g}_{j}}.{{V}_{j}} \end{aligned}$$

where *i* is expressed in binary, $g_{j}$ are its bits, $V_{j}$ are direction numbers, and *⊕* denotes bitwise XOR. This construction minimizes discrepancy in multidimensional sampling.

#### Halton

The Halton sequence is represented in Eq. (13) built with coprime bases and provides a deterministic method of uniformly scattered points on dimensions^5^.

13 $$\begin{aligned} {{H}_{b}}(i)=\sum \limits _{j=0}^{M}{\frac{{{\mathbb {R}}_{j}}}{{{\mathbb {C}}^{j+1}}}} \end{aligned}$$

Where, $i = \mathbb {R}_{0} + \mathbb {R}_{1}\mathbb {C}$ $+ \mathbb {R}_{2}\mathbb {C}^{2} + \dots + \mathbb {R}_{M}\mathbb {C}^{M}$ is the expansion of *i* in base $\mathbb {b}.$

#### Sierpinski

The Sierpinski initialization in Eq. (14) provides a fractal distribution, that leads to better coverage of the search space by the recursive division of the range into smaller sub areas^6^.

14 $$\begin{aligned} {{Z}_{i}}=\frac{{{\mathbb {Q}}_{i}}}{{{2}^{m}}},i=1,2,....,N \end{aligned}$$

Where, ${{\mathbb {Q}}_{i}}$ is an integer chosen according to the Sierpinski pattern, and *m* controls the recursion depth. Together, these four initialization strategies provide a more diverse starting population for the proposed search process.

### Local refinement via Great Deluge Algorithm

GDA is used in the proposed method as a local refinement and adaptive acceptance mechanism. It is often compared with SA, but instead of using temperature as the acceptance barrier, GDA uses an upper boundary known as the water level^4^. Better solutions are always accepted, while some non improving solutions may also be accepted if their objective value remains within the current water level limit. This controlled acceptance supports more flexible local refinement.

Compared with SA, GDA is simpler in terms of parameter handling because it mainly depends on the water level setting and its decay behaviour^45,46^. In minimization problems, a candidate solution can be retained if its objective value is less than or equal to the current water level. As the search continues, the water level is updated gradually to control the acceptance of candidate solutions during local refinement^47^. This makes GDA useful for improving local search while still allowing limited exploration in the earlier iterations.

We adopt an adaptive GDA for local refinement in the proposed method. The initial water level is set based on the best initial objective value and the variation in the initial population. The initial water level is defined as:

15 $$\begin{aligned} W{{L}_{0}}=f({{\chi }_{best,0}})+\tau .{{\gamma }_{f,0}} \end{aligned}$$

Where ${{\gamma }_{f,0}}$ is the standard deviation, ${{\chi }_{best}}$ denotes the best objective value among the initial solutions.

The water level is then linearly decreased over the course of iterations, with a decay rate defined as:

16 $$\begin{aligned} decayRate=\frac{W{{L}_{0}}-{{\chi }_{best}}}{{{T}_{\max }}} \end{aligned}$$

Where ${{T}_{\max }}$ is the maximum number of iterations. The water level is gradually reduced at each iteration as:

17 $$\begin{aligned} W{{L}_{t+1}}=W{{L}_{t}}-decayRate \end{aligned}$$

Candidate solutions *χ '* is accepted when

18 $$\begin{aligned} f(\chi ')\le W{{L}_{t}}+{{\rho }_{t}} \end{aligned}$$

where the adaptive slack ${{\rho }_{t}}$ decays over time and scales with the local quality gap.

19 $$\begin{aligned} {{\rho }_{t}}={{\rho }_{0}}\exp (-\beta t)(\chi _{best}^{t}-\overline{{{\chi }^{t}}}) \end{aligned}$$

Here, ${{\rho }_{0}}$ is the initial slack *β >0* is a decay factor, $\chi _{best}^{t}$ is the best solution at iteration *t*, and $\overline{{{\chi }^{t}}}$ is the mean objective of the current population. We determined the adaptive slack parameters namely the initial slack $\rho _0$ and the decay factor *β* through empirical trials across our benchmark images. This tuning was necessary to smoothly shift the algorithm from global exploration to local exploitation. The value of *β* determines how fast the constraints become tighter. However, the overall role of the adaptive slack is to avoid getting trapped in local minima for the problem. As the value of the slack is defined by the local gap between the best and mean solution $(\chi _{best}^{t}-\overline{\chi ^{t}})$, the mechanism automatically loosens the requirements if the individuals disperse or approach a local minimum. For a short time during which the algorithm becomes more lenient, the GDA can accept suboptimal solutions and acquire the motivation to get out of the local minima before the strict requirements come into play during the last steps. The generation of new individuals happens according to a diminishing step size:

20 $$\begin{aligned} \chi '=\chi +{{s}_{t}}.\eta , {{s}_{t}}={{s}_{0}}{{(1-\frac{t}{{T}_{\max }})}^{\lambda }} \end{aligned}$$

Where *η* is a random perturbation vector, ${{s}_{0}}$ is the initial step size, *λ >0* controls the decay profile. The algorithm iterates until a maximum number of iterations is reached or no significant improvement is observed, ultimately returning the best solution found.

It should be noted that the GDA acceptance scheme is performed for the entire population during each cycle of the algorithm, but the actual evaluation of this scheme works on a per agent basis. In particular, for each new solution created by any hummingbird within the process of exploration of either the guided foraging behavior or the territorial one, this solution will be evaluated with respect to the current value of the water level in GDA.

**Fig. 2:**
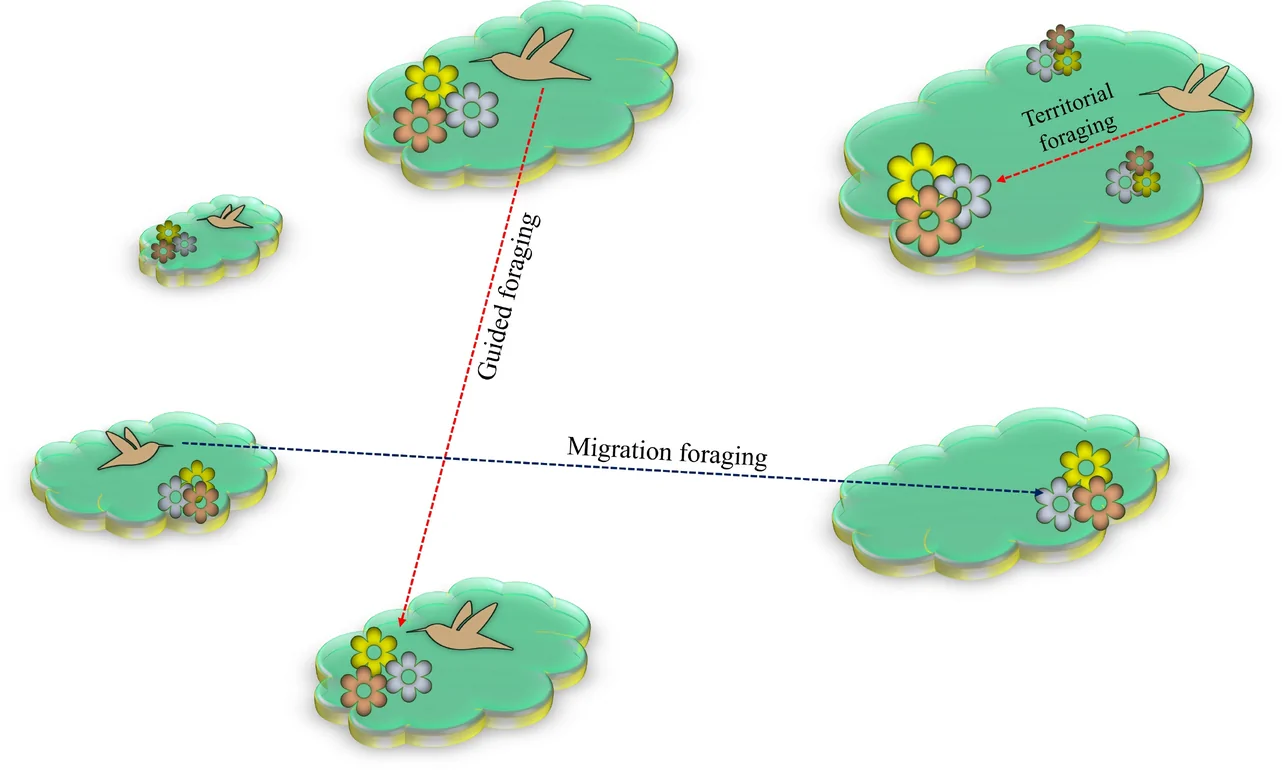
Foraging behaviors of the Artificial Hummingbird Algorithm.

### Proposed artificial hummingbird based energy curve driven multilevel thresholding

In this section a bio inspired optimization algorithm, AHA, built on intelligent behaviors of hummingbirds is introduced^3^. The proposed method is utilized as a resilient and biologically inspired optimizer to tackle the multilevel threshold issue. The initially proposed technique draws inspiration from the intelligent foraging behavior of hummingbirds, their remarkable memory, and their diverse flight approaches incorporating the three primary components of AHA^3^.

#### Biological modeling and core components of AHA

The AHA simulates the foraging behaviour of hummingbirds and organizes the optimization process using three main components: food sources, hummingbirds, and the visit table.

#### Food sources

In AHA, each food source represents a candidate solution. For multilevel image thresholding, this solution is a set of threshold values used to divide the image intensity levels. The nectar refill rate of a food source is represented by its fitness value, where a better fitness value indicates a better threshold solution.

#### Hummingbirds

Each hummingbird is linked with a food source and uses memory to guide its movement. It can remember the quality of food sources and the time elapsed since each source was visited. This helps the search avoid repeated movement around the same region.

#### Visit table

The visit table records the visit status of each food source for every hummingbird. Sources that have not been visited for a longer time are given more attention, and when several sources have the same visit level, the one with better fitness is selected. The visit table is updated at every iteration to maintain a balance between exploration and exploitation. The AHA is used as the main swarm based optimizer for multilevel threshold selection. Based on the foraging behaviour and flight patterns of hummingbirds shown in Fig. 2, AHA applies three search strategies: guided foraging, territorial foraging, and migration foraging. These strategies help coordinate global exploration and local exploitation during the segmentation process.

**Fig. 3:**
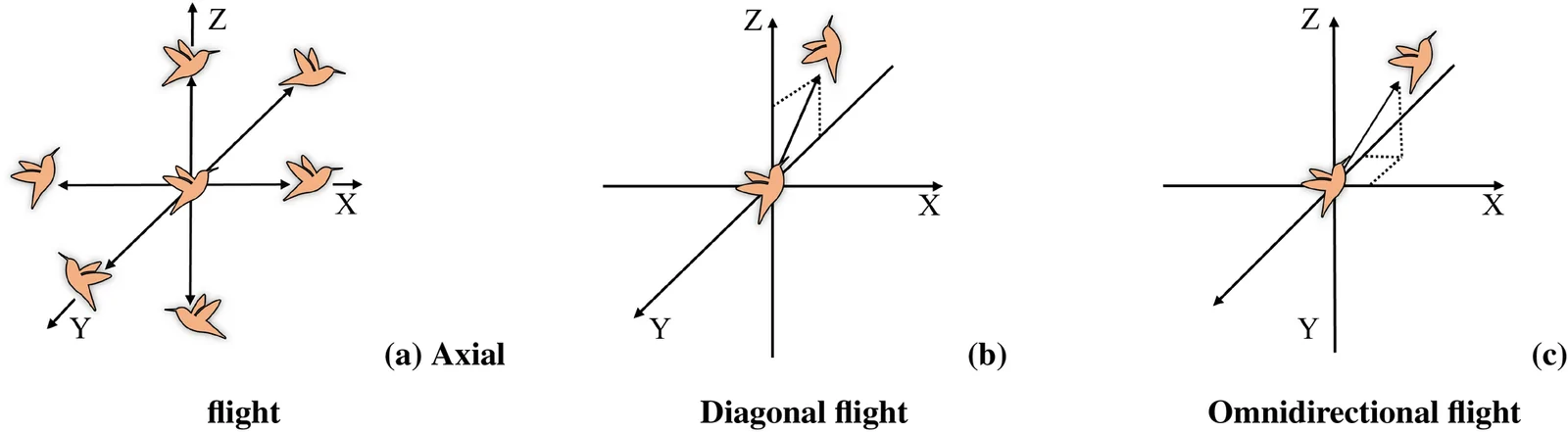
Three hummingbird flight behaviors: (**a**) Axial flight, (**b**) Diagonal flight, and (**c**) Omnidirectional flight.

### Guided foraging

In guided foraging, a hummingbird selects a target food source by considering both the visit history and the nectar quality of available sources. In the optimization process, this means that the agent moves toward a promising solution while also considering sources that have not been visited recently. This behaviour helps the algorithm explore useful regions of the search space without repeatedly focusing on the same solution.

AHA uses a directional switch vector to three different flight patterns: axial, diagonal, and omnidirectional flights, as shown in Fig. 3. These flight patterns control the movement direction of the hummingbird in the *D*-dimensional search space. Axial flight allows movement along one dimension, diagonal flight allows movement along selected dimensions, and omnidirectional flight allows movement along all dimensions. The Eq. (21) defines the axial flight.

21 $$\begin{aligned} d^{i} = {\left\{ \begin{array}{ll} 1, & \text {if } i = \texttt {randi}([1,D]) \\ 0, & \text {otherwise} \end{array}\right. } \quad \text {for } i = 1, \ldots , D \end{aligned}$$

The diagonal flight is defined in Eq. (22).

22 $$\begin{aligned} d^{i} = {\left\{ \begin{array}{ll} 1, & \text {if } i = p(j), j \in [1, k], \\ & \quad p = \texttt {randperm}(k), k \in \left[ 2, \big \lceil r_1 \cdot (D-2) \big \rceil + 1 \right] \\ 0, & \text {otherwise} \end{array}\right. } \end{aligned}$$

for *i = 1, … , D*.

According to Eq. (23), omnidirectional flight is defined.

23 $$\begin{aligned} d^{i} = 1, \quad \text {for } i = 1, \ldots , D \end{aligned}$$

Using these flight patterns, the $i^{th}$ hummingbird generates a candidate solution around the selected target food source. The updated candidate position is calculated as:

24 $$\begin{aligned} & {{U}_{i}}(t+1)={{x}_{i,target}}(t)+A.d.({{x}_{i}}(t)-{{x}_{i,target}}(t)) \end{aligned}$$

25 $$\begin{aligned} & A \sim \mathscr {N}(0,1) \end{aligned}$$

where $x_i(t)$ is the current position of the $i^{th}$ hummingbird at iteration *t*, $x_{i,target}(t)$ is the selected target food source, *A* is a normally distributed random variable with mean 0 and standard deviation 1, and *d* controls the active movement direction. The generated candidate solution is compared with the current solution using the objective function. The position update is defined as:

26 $$\begin{aligned} {{x}_{i}}(t+1)=\left\{ \begin{array}{ll} {{x}_{i}}(t), & \text {if } f({{x}_{i}}(t)) \le f({{U}_{i}}(t+1)) \\ {{U}_{i}}(t+1), & \text {if } f({{x}_{i}}(t)) > f({{U}_{i}}(t+1)) \end{array} \right. \end{aligned}$$

where *f(.)* denotes the objective function to be minimized. Thus, the candidate solution is accepted only when it improves the current fitness value.

#### Visit table management and target selection

The visit table guides the foraging activity of hummingbirds by recording the visit status of each food source. A higher visit value indicates that a food source has not been visited for a longer time, making it more attractive for selection. At each iteration, each hummingbird first considers the food sources with the highest visit level. If more than one source has the same visit level, the source with the better nectar refilling rate, represented by the minimum objective function value, is selected as the target for guided foraging as given in Eq. (25).

After guided foraging, the visit levels of the accessible food sources are increased by one, while the selected target source is reset to zero. If the newly generated candidate solution does not improve the fitness value, the hummingbird retains its current position. However, if a better candidate solution is obtained, it replaces the previous food source, and the corresponding visit table entries are updated. In this case, the new food source will get more visitation preference among the other hummingbirds visitation table to encourage exploration and maintain diversity among the population.

**Fig. 4:**
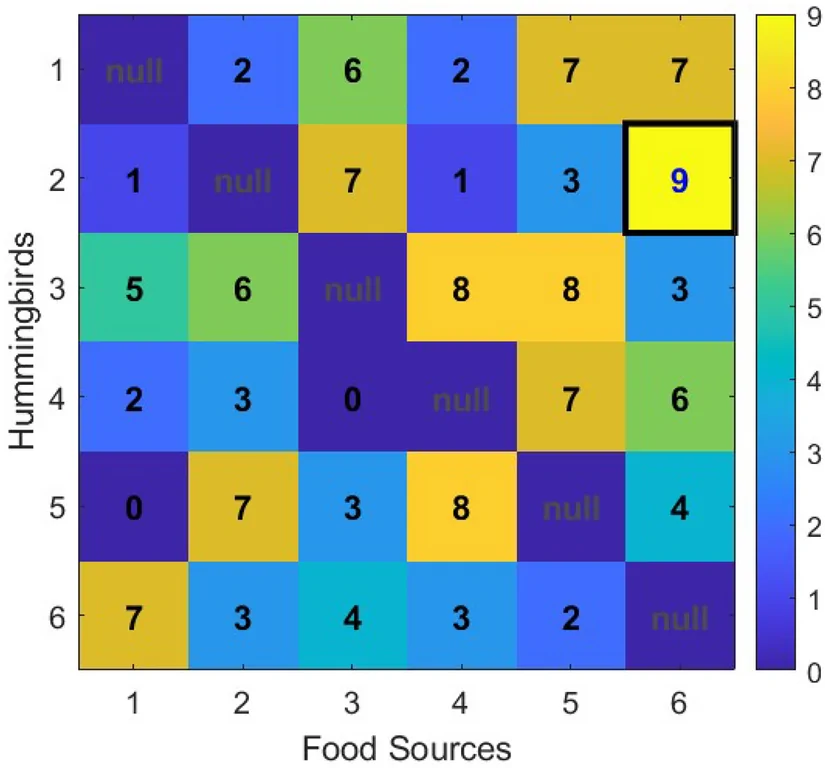
Foraging visit table of six hummingbirds.

Figure 4 shows the visitation table with six food sources where each food source has only one hummingbird at the moment. Each cell in the table represents visitation values; this means that the value represents the number of time intervals passed after a particular food source was visited by the respective hummingbird. For example, in the Fig. 4 the cell with the blue border marked with 9 implies that hummingbird 2 has not visited food source 6 for nine time intervals.

The six hummingbirds image shown in Fig. 4, is also used as a test image. This image consists of complicated texture, intensity variation, wing and feather details.

### Territorial foraging

In territorial foraging, a hummingbird prefers to find a new food source once the nectar in its current flower is depleted. It avoids visiting food sources that are already being targeted by other hummingbirds. As a result, a hummingbird may easily relocate to a nearby area inside its own territory, where it might discover a new food supply that is superior to its present one. The following is the mathematical formula that represents the local search for a potential food source by hummingbirds in the territorial foraging strategy:

27 $$\begin{aligned} & {{U}_{i}}(t+1)={{x}_{i}}(t)+B.d.{{x}_{i}}(t) \end{aligned}$$

28 $$\begin{aligned} & B \sim \mathscr {N}(0, 1) \end{aligned}$$

where the normal distribution is applicable to the territorial variable *B*. Exploiting its distinctive aerial capabilities, $\mathscr {N} (0,1)$ in Eq. (28), characterized by a mean of 0 and a standard deviation of 1, enables each hummingbird to identify a novel food source in nearby to its current location. The visit table must be revised subsequent to the execution of the territorial foraging strategy.

### Migration foraging

In AHA, migration foraging represents the ability of a hummingbird to leave a poor food source and move to a more promising region. This behaviour improves global search in multilevel thresholding and helps the algorithm avoid repeated search around weak solutions. When the migration condition is satisfied, the hummingbird associated with the worst food source is relocated to a new position generated within the search space. The visit table is then updated accordingly. The new position is calculated as:

29 $$\begin{aligned} {x}_{\text {worst}}(t+1) = LOW + r \cdot (UP - LOW) \end{aligned}$$

where *r* is a random vector in *[0,1]*, and *LOW* and *UP* represent the lower and upper bounds of the search space.

**Fig. 5:**
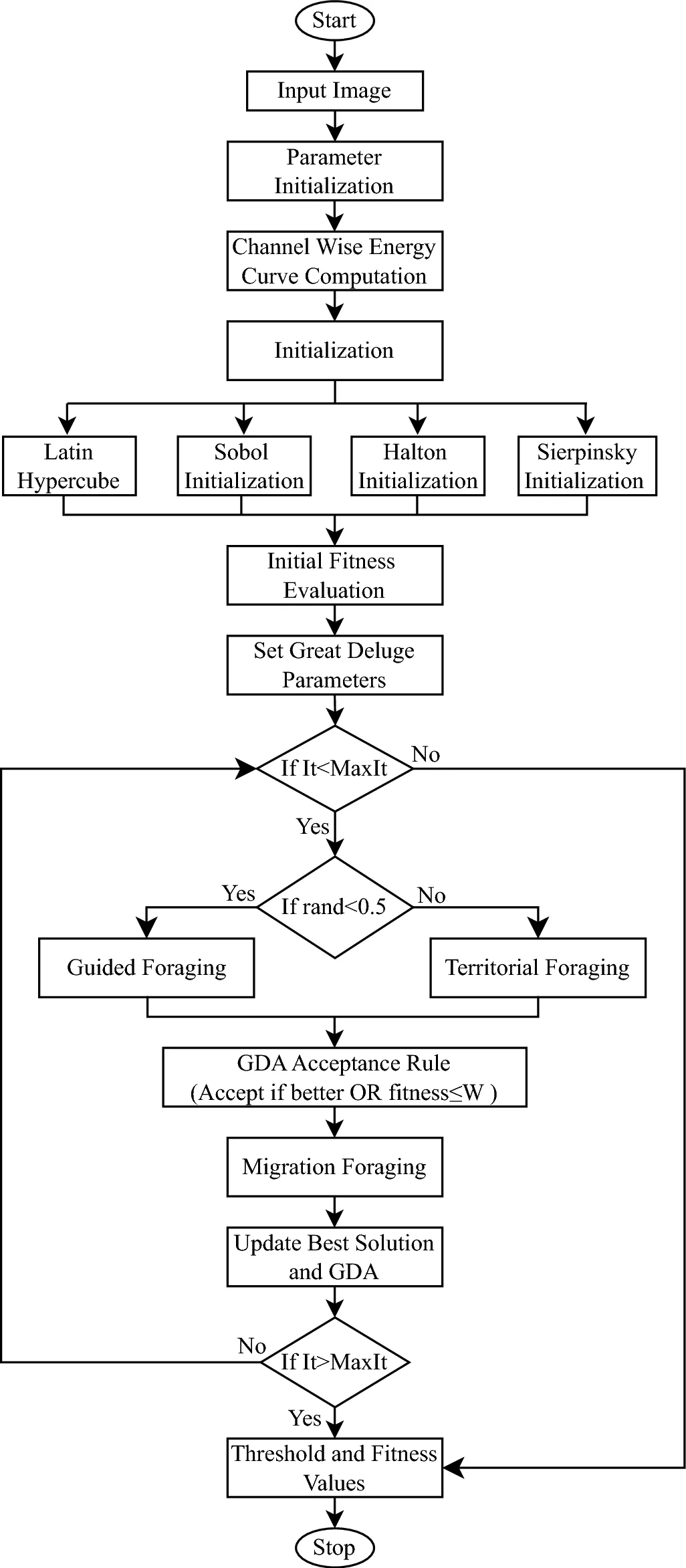
Operational framework of the proposed segmentation method.

Migration helps the algorithm to steer clear of areas that do not provide any gain in terms of the fitness function during either guided or territorial foraging. When a source of food is left unchanged for an extended period of time, the hummingbird associated with that area is motivated to search in a different region. In this way, diversification is fostered, and the danger of getting stuck in local minima or early convergence is reduced. At the same time, in case a good food source has been found, the other birds will be motivated to migrate to that region. Migration takes place after a number of iterations according to Eq. (30). A flowchart of the whole process is provided in Fig. 5.

30 $$\begin{aligned} M=2n \end{aligned}$$

### Computational complexity analysis

The total computational complexity of the proposed hybrid framework is evaluated by analyzing its three primary stages. Let *m × n* represent the image dimensions, *L* be the maximum gray level (256), *C* be the number of color channels, *D* be the number of threshold dimensions, *nPop* be the population size, and *T* be the maximum number of iterations.

**Energy curve computation (Preprocessing):** As detailed in Algorithm 1, this phase evaluates spatial neighborhood interactions for every pixel. For a neighborhood of size *W*, computing the energy for all gray levels across *C* channels requires $O(C \cdot L \cdot m \cdot n \cdot W)$ operations. Since *C* and *W* are small constants, this mathematically simplifies to an asymptotic complexity of $O(L \cdot m \cdot n)$.

**Initialization phase:** Generating the initial population utilizing four structured sequences (Latin Hypercube, Sobol, Halton, and Sierpinski) requires assigning *D* dimensional variables for *nPop* individuals. The complexity for this step is strictly *O(nPop · D)*.

**Optimization and refinement (AHA-GDA):** During the AHA and GDA phase, the algorithm operates over *T* iterations. In each iteration, updating the positions (guided, territorial, or migration foraging) takes *O*(*D*) operations per individual. Furthermore, evaluating the MCEM fitness function for each updated candidate solution requires iterating through the histogram bins, taking *O*(*L*) time. Therefore, the cost per iteration for the entire population is *O(nPop · (D + L))*. Over *T* iterations, this results in an optimization complexity of $O(T \cdot nPop \cdot (D + L))$.

**Total complexity:** By summing the independent phases, the overall mathematical formulation of the algorithm’s time complexity is:

31 $$\begin{aligned} O(L \cdot m \cdot n) + O(nPop \cdot D) + O(T \cdot nPop \cdot (D + L)) \end{aligned}$$

Because initialization *O(nPop · D)* is asymptotically smaller than the iterative optimization step, the final dominant computational complexity simplifies to:

32 $$\begin{aligned} O(L \cdot m \cdot n + T \cdot nPop \cdot (D + L)) \end{aligned}$$

### Mathematical formulation of component synergy and architecture comparison

The integration of multiple hybrid components and prove their mathematical synergy, the state transition of the proposed algorithm can be modeled as a composite function. Let $\mathscr {S}$ represent the search space of possible threshold vectors. The complete synergistic progression of a population state *P* from iteration *t* to *t+1* is defined by the following mapping:

33 $$\begin{aligned} P_{t+1} = \mathscr {G} \left( \mathscr {A}(P_t), WL_t, \textbf{E}^f \right) \end{aligned}$$

This composite formulation mathematically binds the four isolated components:

**Initialization:** At *t=0*, the structured sequences (Latin Hypercube, Sobol, Halton, Sierpinski) map an empty space to a highly uniformly distributed initial population:

34 $$\begin{aligned} P_0 = \mathscr {I}(\mathscr {S}) \end{aligned}$$

**AHA foraging** ($\mathscr {A}$): The AHA acts as the primary search operator, mapping the current population $P_t$ to a set of new candidate solutions, denoted as:

35 $$\begin{aligned} U_i = \mathscr {A}(x_i) \end{aligned}$$

**Spatial energy evaluation (**$\textbf{E}^f$**):** Unlike standard models that evaluate fitness *f*(*x*) using 1D histograms, the spatial energy curve $\textbf{E}^f$ evaluates the structural context of both the candidate solution $U_i$ and the current solution $x_i$.

**GDA refinement (**$\mathscr {G}$**):** The GDA acts as the final synergistic filter, judging the candidate solution $U_i$ against the dynamic water level $WL_t$.

**Comparison: standard AHA vs. proposed hybrid architecture**

The increased complexity of the proposed hybrid architecture is theoretically and empirically justified when compared to the standard AHA mechanism.

In a standard AHA, the candidate acceptance is greedy. A generated candidate $U_i$ replaces the current solution $x_i$ for the next iteration only if it yields a better fitness:

36 $$\begin{aligned} x_{i}(t+1) = {\left\{ \begin{array}{ll} U_i, & \text {if } f_{hist}(U_i) < f_{hist}(x_i) \\ x_i(t), & \text {otherwise} \end{array}\right. } \end{aligned}$$

While computationally simpler, this strict greediness combined with context blind 1D histograms ($f_{hist}$) causes standard AHA to frequently stagnate in local optima when processing highly textured images.

Conversely, the proposed hybrid architecture utilizes spatially aware acceptance criterion governed by the GDA water level:

37 $$\begin{aligned} x_{i}(t+1) = {\left\{ \begin{array}{ll} U_i, & \text {if } f_{EC}(U_i) < f_{EC}(x_i) \text { or } f_{EC}(U_i) \le WL_t \\ x_i(t), & \text {otherwise} \end{array}\right. } \end{aligned}$$

The algorithm allowing acceptance of low quality solutions is allowed temporarily if their value is still below the gradually decreasing $WL_t$, which allows avoiding getting stuck in local optima.

## Results and discussion

In addition to the suggested metrics for assessing segmentation outcomes, this section describes the computer specifications used for operations. Additionally, by using multilevel thresholding, it clarifies how metaheuristics are applied to solve the image segmentation problem.

### Experimental conditions

The proposed method was evaluated using two public datasets: BSDS500^11^ and NASA^12^. The BSDS500 dataset contains 500 natural images, including objects, animals, buildings, vegetation, and outdoor scenes. These images have different textures, object boundaries, backgrounds, and illumination conditions. The NASA Earth Observatory dataset contains 44 satellite images. These images include land, vegetation, water bodies, clouds, deserts, and other earth surface regions. They contain dense spatial details and different colour variations, which make them suitable for testing satellite image segmentation. No preprocessing or annotation procedure was performed on the NASA dataset. The dataset images were directly utilized for experimental evaluation. Since the proposed method is not based on machine learning or deep learning techniques, no dataset splitting into training or testing subsets was required. The complete dataset was directly used for performance evaluation and comparison.

All images taken from BSDS500 and NASA databases were used to perform the experiments. For the sake of brevity, the manuscript presents only representative segmented images in the result outputs, while the values reported in the result tables were obtained from the corresponding segmented images. The experiments were conducted using MATLAB R2023b software on the Windows 11 64-bit operating system equipped with an Intel Core i7 CPU and 16 GB of memory. All methods were tested using three threshold values: *nT = 3*, *nT = 5*, and *nT = 8*. To guarantee the comparability of the results, the same parameters were used for testing all the proposed approaches. The test consisted of 200 iterations with 30 individuals in the population. The color image was divided into the R, G, and B components. These components were processed separately during segmentation and then combined together to create the final segmented image.

### Code availability

The MATLAB code for the proposed method is available in a public GitHub repository: https://github.com/suprajatirumalasetti/AHA_GDA_Image_Segmentation_Code.

### Quantitative evaluation

Evaluating the quality of segmented images has become crucial as image segmentation techniques have expanded in recent years. Seven widely accepted image quality metrics were employed: PSNR^13^, SSIM^14^, FSIM^15^, QILV^16^, Correlation Coefficient^17^, EPI^18^, and MIF^19^.

#### Peak signal to noise ratio

The PSNR metric defines the extent of fidelity achieved by segmenting the image compared to the original image, which is measured in dB. Higher PSNR metrics imply lesser distortion and better retention of image information through the process of image segmentation. Table 3 provides the PSNR measures for BSDS500 and NASA images using thresholds of 3, 5, and 8. It can be seen that the proposed technique generally produces higher PSNR measurements as compared to the other comparative algorithms, indicating the effectiveness of the former in retaining image information through segmentation. This holds true for both types of images (BSDS500 and NASA) and implies that the proposed technique retains information more effectively during segmentation. This inference is substantiated by the fact that the proposed technique outperforms all other baseline techniques.

**Fig. 6:**
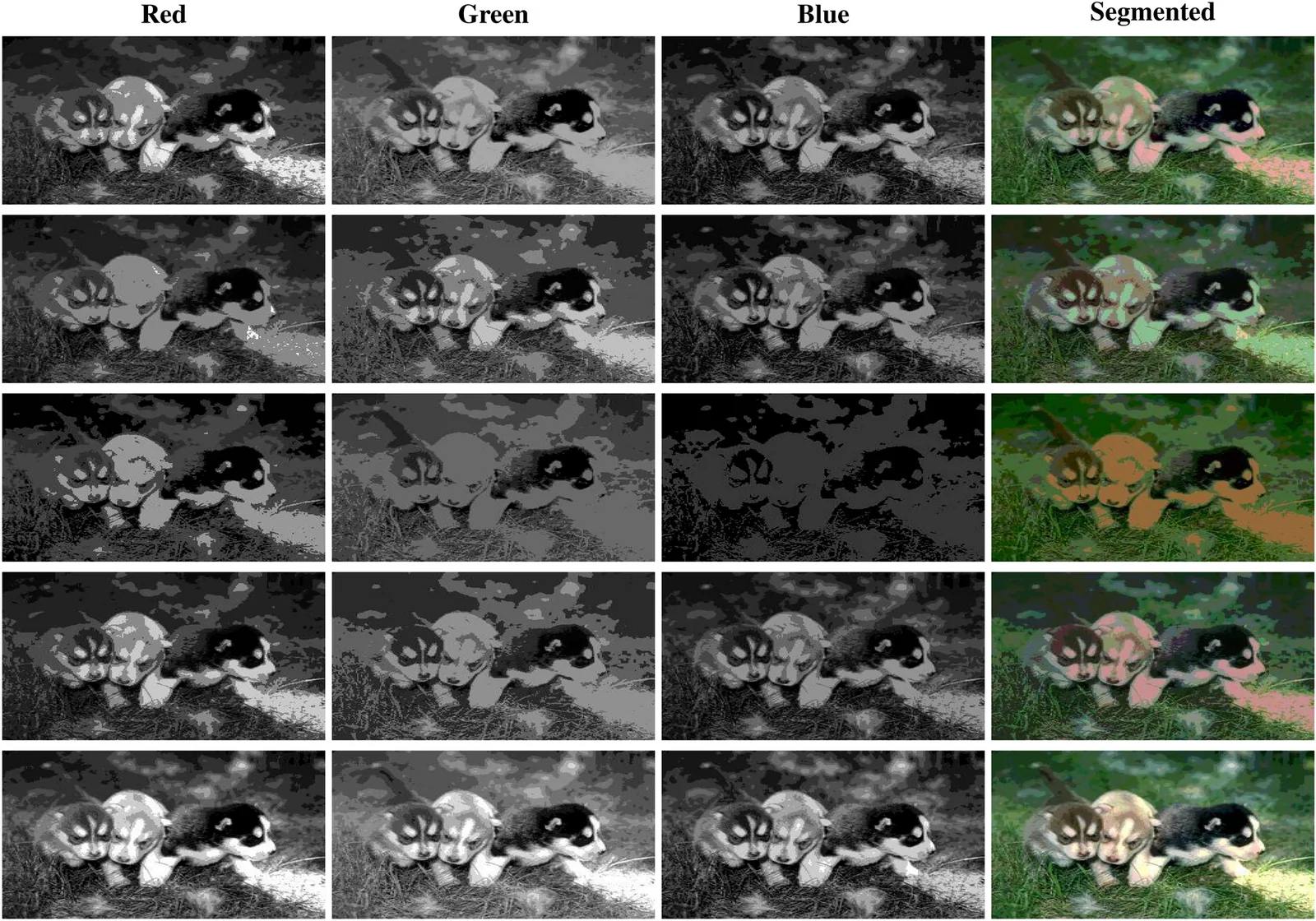
Comparison of AO, EO, PSO, WOA, and proposed method from top to bottom for Red, Green, Blue channel thresholding and final segmentation results for the image 247003 of 8 level threshold from left to right.

**Fig. 7:**
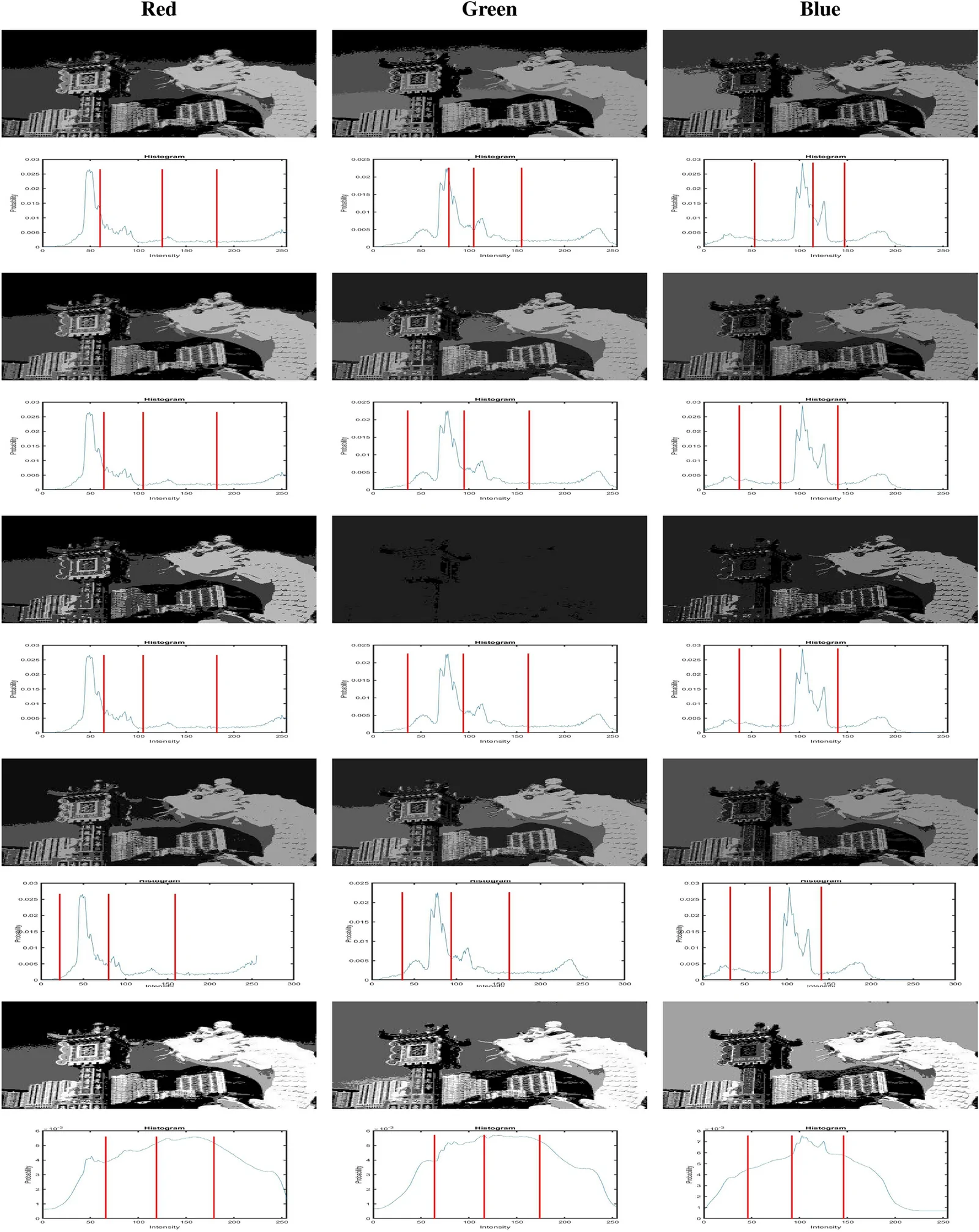
Comparison of 120093 three level threshold with segmented and histogram images with AO, EO, PSO, WOA, and proposed method from top to bottom.

**Table 3:** Comparative PSNR results on BSDS500 and NASA datasets with varying threshold levels.

BSDS500							NASA							
Image name	nT	AO	EO	PSO	WOA	Proposed	Image name	T	AHA	AO	EO	PSO	WOA	Proposed
120093	3	24.7293	24.5748	24.3999	24.5541	**30.4735**	Kizimen	3	33.9567	31.7256	32.0691	33.3350	33.8267	**33.9567**
	5	25.3307	24.9584	24.7841	25.3966	**33.6325**		5	36.3224	32.5610	33.9732	35.8277	33.7256	**36.3300**
	8	25.4443	25.1349	24.9965	24.8316	**34.6113**		8	37.2355	29.7386	37.6044	38.3972	31.2038	**38.4143**
159002	3	25.5404	25.6349	25.3354	25.6329	**29.3680**	Yellowstone	3	33.1830	36.6818	33.9769	36.0187	34.0118	**33.5834**
	5	26.0633	26.8489	25.6880	26.2479	**30.1126**		5	35.5804	31.5491	32.2577	33.3947	31.3954	**35.7575**
	8	26.9512	26.9600	25.5164	25.9203	**31.3913**		8	40.1354	36.5936	30.3099	39.5882	40.3297	**40.3536**
189029	3	25.5069	25.4185	25.2380	25.3996	**28.9393**	Goldpanfire	3	35.1019	30.6552	34.7464	32.5210	30.9620	**35.1292**
	5	25.8958	26.2386	25.4571	25.7588	**29.5218**		5	35.0242	32.1257	33.4889	32.0708	32.3470	**36.2502**
	8	26.3641	26.5805	25.4159	26.6839	**30.3501**		8	39.2726	36.8185	33.9164	27.7371	31.1206	**39.4828**
228076	3	25.3650	25.3994	25.2321	25.4777	**30.7777**	Nyamuragira	3	31.5429	30.1476	30.2806	29.6422	30.3468	**31.6743**
	5	26.2136	25.8471	25.5025	25.9352	**32.5953**		5	35.1350	34.8388	27.3618	31.9180	31.1153	**35.2226**
	8	26.5935	26.4066	25.5833	26.2526	**36.2204**		8	36.8788	31.1302	27.2606	35.2393	27.1082	**37.3473**
247003	3	25.1108	25.0639	24.6049	25.1223	**30.2612**	Rockhouse	3	35.1465	30.7116	34.8418	31.9100	32.3914	**35.1465**
	5	25.9810	25.7177	24.9926	25.4676	**33.1653**		5	37.5957	28.5174	36.3228	36.6080	35.5939	**37.8557**
	8	26.0792	25.6968	24.6945	25.6616	**35.9528**		8	39.9944	33.7626	34.0210	39.3624	32.9135	**40.3916**
326085	3	25.1668	25.0005	24.7798	25.0315	**31.0340**	Yarrabin	3	33.0126	32.2753	31.4749	32.3587	30.9591	**33.0856**
	5	25.5039	25.5575	24.9379	25.5555	**33.2250**		5	34.6206	32.0504	32.2231	34.5591	31.1201	**34.6871**
	8	26.0960	25.7938	25.0069	25.6344	**36.5538**		8	39.9298	28.0078	32.4493	37.9832	25.9390	**39.9216**

Significant values are in [bold].

**Table 4:** Comparative SSIM results on BSDS500 and NASA datasets at varying threshold levels.

BSDS500							NASA							
Image name	nT	AO	EO	PSO	WOA	Proposed	Image name	nT	AHA	AO	EO	PSO	WOA	Proposed
120093	3	0.9605	0.9595	0.8916	**0.9627**	0.9570	Kizimen	3	**0.9491**	0.9281	0.9423	0.8976	0.9410	0.9491
	5	0.9797	0.9771	0.9274	0.9811	**0.9864**		5	0.9756	0.9595	0.9571	0.8866	0.9450	**0.9763**
	8	0.9858	0.9805	0.9275	0.9671	**0.9947**		8	0.9892	0.9873	0.9547	0.8733	0.9614	**0.9905**
159002	3	0.9679	**0.9762**	0.8674	0.9761	0.9748	Yellowstone	3	**0.9561**	0.8866	0.8947	0.7897	0.8935	0.9561
	5	0.9839	0.9855	0.9421	0.9828	**0.9897**		5	0.9800	**0.9845**	0.9197	0.8540	0.9395	0.9803
	8	**0.9912**	0.9866	0.9108	0.9794	0.9943		8	0.9901	0.9863	0.9751	0.8406	0.9244	**0.9903**
189029	3	0.9710	0.9751	0.8706	0.9761	**0.9816**	goldpanfire	3	0.9519	0.9508	0.9385	0.9159	0.9020	**0.9530**
	5	0.9845	0.9865	0.8736	0.9874	**0.9904**		5	0.9783	0.9509	0.9440	0.8329	0.9528	**0.9783**
	8	0.9912	0.9870	0.8835	0.9904	**0.9953**		8	0.9880	0.9812	0.9522	0.8637	0.9636	**0.9888**
228076	3	0.9725	**0.9776**	0.9082	0.9710	0.9755	Nyamuragira	3	0.9543	0.9380	0.9427	0.8794	0.9471	**0.9683**
	5	0.9877	0.9849	0.9204	0.9884	**0.9910**		5	**0.9861**	0.9654	0.9704	0.8802	0.9642	0.9861
	8	0.9844	**0.9901**	0.9632	0.9848	0.9937		8	0.9915	0.9911	0.9636	0.8820	0.9773	**0.9937**
247003	3	0.9670	0.9702	0.897	**0.9725**	0.9629	Rockhouse	3	**0.9586**	0.9404	0.9548	0.9105	0.9492	**0.9586**
	5	0.9825	0.9814	0.9380	0.9833	**0.9882**		5	0.9763	**0.9833**	0.9667	0.9224	0.9701	0.9764
	8	0.9895	0.9839	0.9068	0.9776	**0.9950**		8	0.9879	0.9849	0.9712	0.8830	0.9529	**0.9879**
326085	3	0.9703	0.9670	0.9009	0.9682	**0.9759**	Yarrabin	3	**0.9693**	0.9644	0.9441	0.9188	0.9452	**0.9693**
	5	0.9803	0.9817	0.9037	0.9839	**0.9891**		5	0.9831	0.9807	0.9572	0.8496	0.9443	**0.9834**
	8	0.9888	0.9849	0.9329	0.9847	**0.9946**		8	0.9914	0.9802	0.9650	0.8918	0.9656	**0.9917**

Significant values are in [bold].

**Table 5:** FSIM comparison results for different algorithms on BSDS500 and NASA dataset images.

BSDS500							NASA							
Image name	nT	AO	EO	PSO	WOA	Proposed	Image name	T	**AHA**	AO	EO	PSO	WOA	Proposed
120093	3	0.7917	**0.7994**	0.6792	0.7937	0.7583	Kizimen	3	**0.8048**	0.7610	0.7995	0.5680	0.7866	0.8048
	5	0.8228	0.8248	0.7187	0.8231	**0.8408**		5	0.8998	0.8452	0.8608	0.5719	0.7925	**0.9011**
	8	0.8547	0.8363	0.6758	0.8113	**0.8971**		8	0.9147	0.9265	0.8408	0.5298	0.8343	**0.9544**
159002	3	0.6909	**0.7184**	0.5817	0.7189	0.7004	Yellowstone	3	0.8050	0.7317	0.7552	0.5829	0.7292	**0.8060**
	5	0.7709	0.7903	0.7034	0.7597	**0.8129**		5	0.8968	0.8862	0.7917	0.6183	0.8457	**0.8988**
	8	0.8428	0.7891	0.6015	0.7437	**0.8936**		8	0.9455	0.9289	0.8907	0.5949	0.7919	**0.9537**
189029	3	0.7378	0.7602	0.6785	**0.7637**	0.7472	Goldpanfire	3	0.8034	0.7760	0.8005	0.7011	0.7081	**0.8074**
	5	0.7967	0.8095	0.7025	**0.8176**	0.8202		5	**0.9014**	0.8350	0.8033	0.4821	0.8456	0.8992
	8	0.8518	0.8229	0.6831	0.8388	**0.8805**		8	0.9543	0.9232	0.8300	0.5764	0.8556	**0.9551**
228076	3	0.8299	**0.8388**	0.7624	0.8185	0.7935	Nyamuragira	3	0.6945	0.6379	0.6359	0.5998	0.6156	**0.6955**
	5	0.8502	0.8674	0.8013	**0.8777**	0.8663		5	**0.7951**	0.7157	0.7217	0.5379	0.6592	0.7948
	8	0.8820	0.8752	0.7882	0.8502	**0.9053**		8	0.8821	0.8410	0.7385	0.5977	0.7257	**0.8824**
247003	3	0.6911	0.6901	0.5581	**0.6939**	0.6406	Rockhouse	3	0.7512	0.7091	0.7468	0.5575	0.7294	**0.7650**
	5	0.7401	0.7455	0.6230	0.7479	**0.7651**		5	**0.8760**	0.8630	0.8187	0.5620	0.8255	0.8756
	8	0.8080	0.7544	0.5624	0.7182	**0.8599**		8	0.9310	0.9002	0.8194	0.4628	0.7163	**0.9406**
326085	3	0.7114	0.6967	0.5808	0.7072	**0.7105**	Yarrabin	3	0.7120	0.7128	0.7015	0.5693	0.6877	**0.7320**
	5	0.7692	0.7973	0.6272	0.8115	**0.8355**		5	**0.8556**	0.8194	0.7607	0.4425	0.7059	0.8539
	8	0.8477	0.8319	0.6308	0.8045	**0.9020**		8	0.9326	0.8656	0.8001	0.4558	0.8157	**0.9334**

Significant values are in [bold].

**Table 6:** QILV comparison results for different algorithms on BSDS500 and NASA dataset images.

BSDS500							NASA							
Image name	nT	AO	EO	PSO	WOA	Proposed	Image name	nT	AHA	AO	EO	PSO	WOA	Proposed
120093	3	0.9141	**0.9187**	0.5782	0.9141	0.9094	Kizimen	3	**0.8456**	0.8304	0.8302	0.8020	0.8367	0.8456
	5	0.9552	0.9554	0.4666	0.9166	**0.9610**		5	0.9170	0.8820	0.8805	0.8115	0.8407	**0.9196**
	8	0.9843	0.9589	0.7926	0.9429	**0.9853**		8	0.9620	0.9580	0.8660	0.8081	0.9079	**0.9677**
159002	3	0.8902	0.8923	0.6088	0.8919	**0.9270**	Yellowstone	3	0.8138	0.6788	0.6917	0.5812	0.6722	**0.8149**
	5	0.9479	0.9362	0.6521	0.9175	**0.9768**		5	0.9051	0.9266	0.7330	0.7254	0.7944	**0.9063**
	8	0.9753	0.9414	0.7246	0.9310	**0.9834**		8	0.9573	0.9441	0.8986	0.6763	0.7627	**0.9592**
189029	3	0.9327	0.9237	0.6133	0.9248	**0.9686**	Goldpanfire	3	0.8327	**0.8473**	0.8155	0.7470	0.7188	0.8327
	5	0.9414	0.9472	0.5299	0.9502	**0.9815**		5	0.9121	0.8483	0.8020	0.7273	0.8459	**0.9136**
	8	0.9692	0.9695	0.4418	0.9362	**0.9930**		8	0.9558	0.9482	0.8295	0.5668	0.8794	**0.9567**
228076	3	0.8235	0.9133	0.3386	0.9111	**0.9231**	Nyamuragira	3	0.9115	0.8513	0.8580	0.7724	0.8683	**0.9125**
	5	0.9537	0.9493	0.4164	0.9376	**0.9711**		5	0.9579	0.9224	0.9459	0.8067	0.9096	**0.9583**
	8	0.9529	0.9548	0.5755	0.9249	**0.9758**		8	0.9828	**0.9831**	0.9226	0.8641	0.9571	0.9813
247003	3	**0.9202**	0.9122	0.8165	0.8854	0.8990	Rockhouse	3	0.8668	0.8214	0.8599	0.8186	0.8324	**0.8688**
	5	**0.9607**	0.9397	0.8660	0.9382	0.9636		5	0.9199	**0.9607**	0.9004	0.8585	0.9018	0.9206
	8	0.9745	0.9399	0.7787	0.9497	**0.9836**		8	0.9558	0.9451	0.8968	0.6026	0.8598	**0.9567**
326085	3	0.9008	0.8991	0.6494	0.8988	**0.9678**	Yarrabin	3	0.9003	0.8878	0.8227	0.8253	0.8118	**0.9009**
	5	0.9431	0.9414	0.7734	0.9402	**0.9800**		5	0.9390	**0.9508**	0.8507	0.8278	0.8137	0.9401
	8	0.9797	0.9412	0.1583	0.9664	**0.9874**		8	0.9706	0.9287	0.8733	0.8246	0.9002	**0.9736**

Significant values are in [bold].

**Fig. 8:**
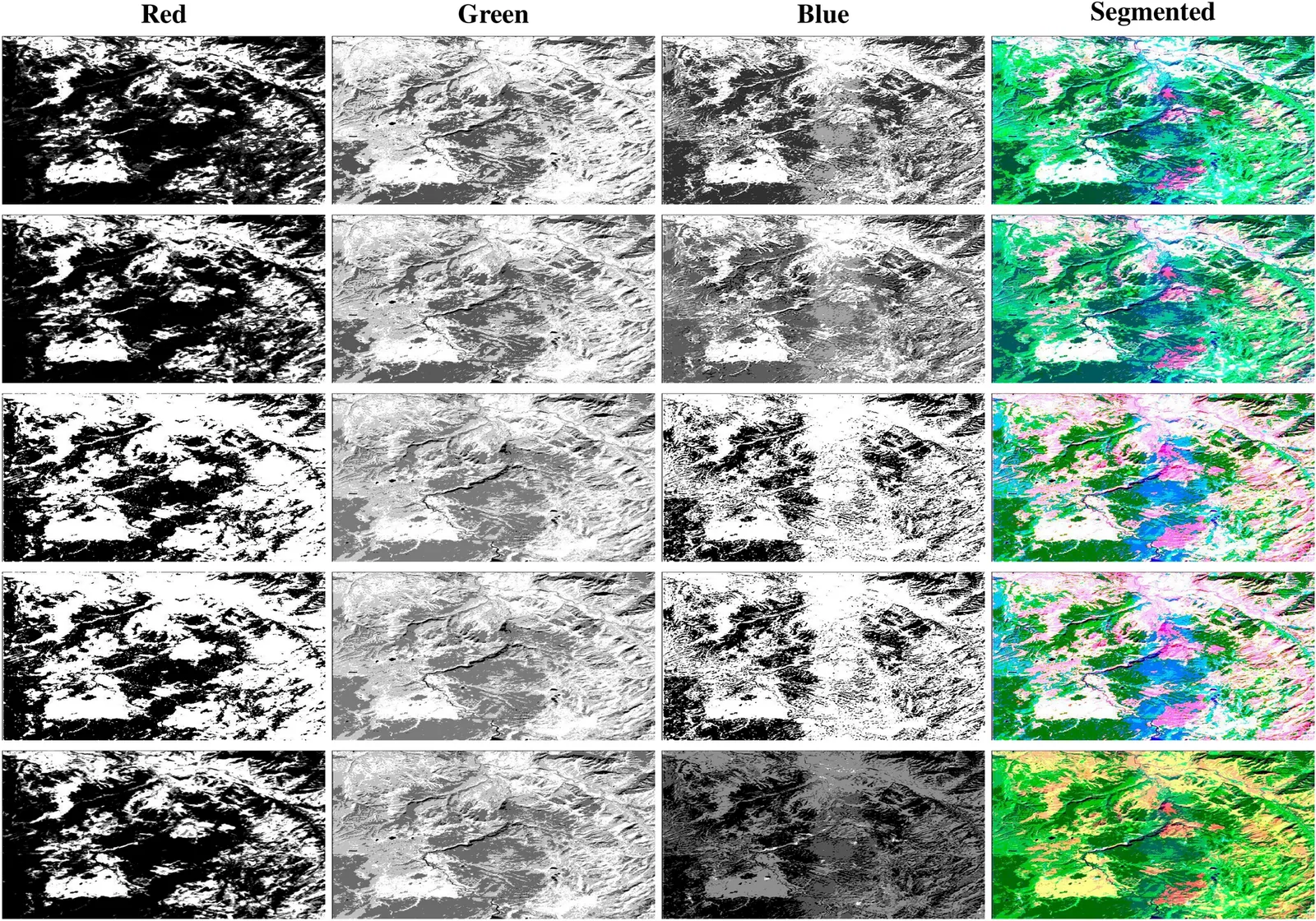
Comparison of AO, EO, PSO, WOA, and proposed method from top to bottom for Red, Green, Blue channel thresholding and final segmentation results for the image Yellowstone of 3 level threshold from left to right.

**Fig. 9:**
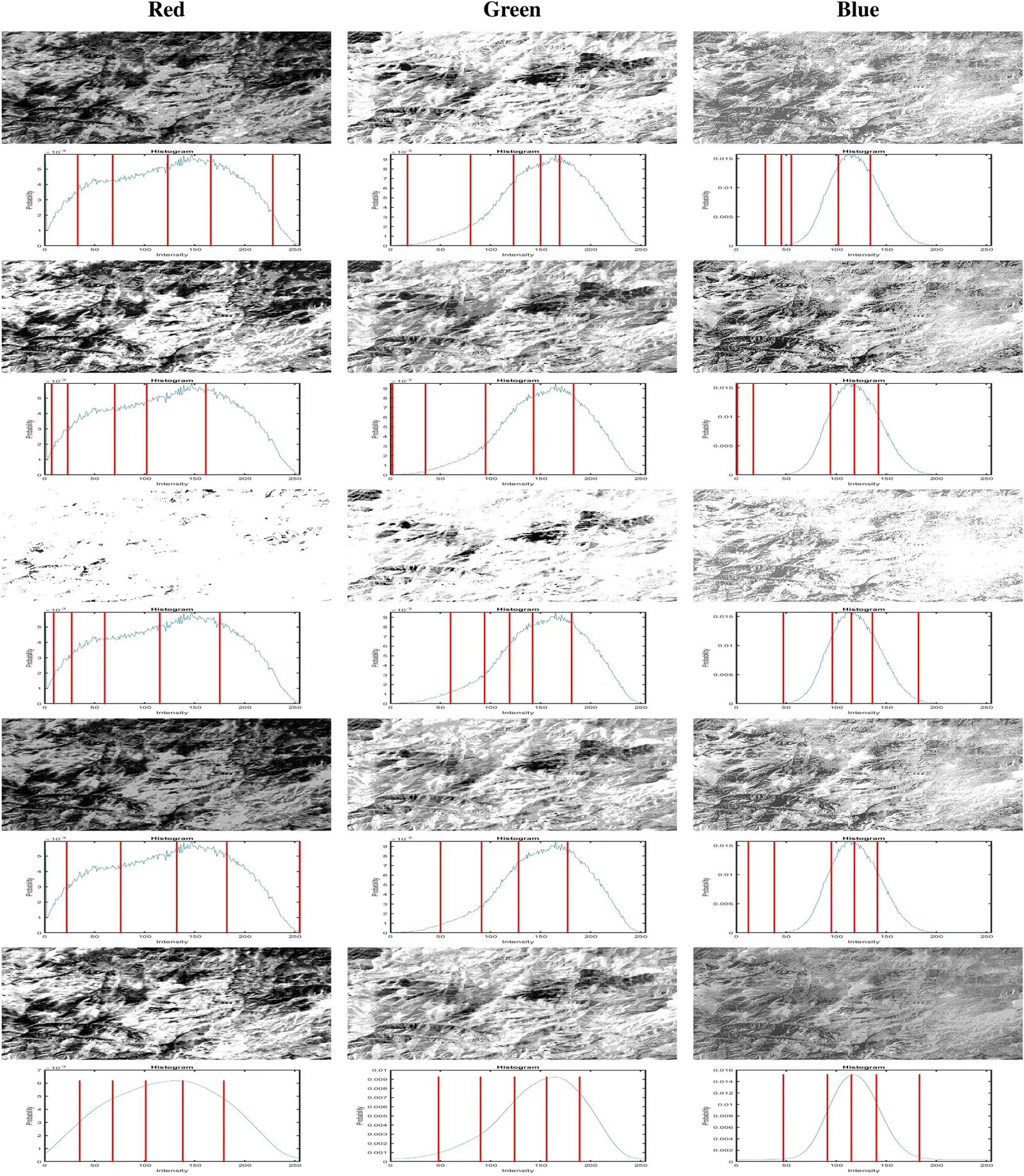
Comparison of Goldpanfire five level threshold with segmented and histogram images with AO, EO, PSO, WOA and proposed from top to bottom, and Proposed method with segmented images and energy curve.

#### Structural similarity index measure

SSIM was used to evaluate how well the segmented image preserves the structural information of the original image. Which takes into account not only pixel-to-pixel comparison but also considers luminance, contrast, and structure of the compared images. Therefore, SSIM can be regarded as the metric for verifying whether the segmentation algorithm is able to preserve structure of the initial image. According to Table 4, the values obtained by the proposed segmentation algorithm for both BSDS500 and NASA datasets are quite consistent regardless of the threshold value used. This suggests that the proposed method preserves structural information in both natural and satellite images with various boundary and texture features.

#### Feature similarity index measure

FSIM was utilized as a measure for feature level analysis to identify whether segmentation has retained any key visual features from the original image. Unlike pixel level analysis, FSIM uses perceptually significant information in its calculation, including phase congruency and gradient magnitude. Hence, FSIM is better suited for analyzing the quality of edge, texture, and local feature preservation by multilevel thresholding. The FSIM results reported in Table 5 provide a feature-level insight into the quality of segmentation results. As can be seen from the table, the new technique produces consistent results irrespective of the threshold level used and does not experience as steep a drop compared to some other baseline techniques. Therefore, we may conclude that the introduced approach is more efficient at preserving local visual features especially in textured images.

#### Quality index based on local variance

The local variance acts as the index that will determine how well small variations and local intensities have been retained after segmentation. Therefore, in this research, the QILV has been used as the evaluation metric. The higher the value of QILV, the better the ability of the segmented image to retain local variations. In multilevel thresholding, the loss of local variations is a major problem. Therefore, the higher value of QILV shows the strength of this approach to preserve local variations even with the use of high thresholds. As shown in Table 6, the proposed method has performed better as compared to the other methods. This performance increases with the increase in the threshold while other methods show deterioration in performance.

**Table 7:** Correlation comparison results for different algorithms on BSDS500 and NASA dataset images.

BSDS500							NASA							
Image name	nT	AO	EO	PSO	WOA	Proposed	Image name	nT	AHA	AO	EO	PSO	WOA	Proposed
120093	3	0.7146	0.7391	0.4135	0.7255	**0.7497**	Kizimen	3	0.5611	0.4859	0.5575	0.2563	0.5250	**0.5613**
	5	0.7657	0.7493	0.3500	0.7423	**0.8335**		5	0.6949	0.5609	0.6408	0.2952	0.5340	**0.6929**
	8	0.8596	0.7835	0.4353	0.7598	**0.8858**		8	0.7697	0.6993	0.5933	0.2913	0.5322	**0.7727**
159002	3	0.4799	0.5872	0.3401	0.5961	**0.6310**	Yellowstone	3	0.6304	0.4680	0.5838	0.2938	0.5868	**0.6287**
	5	0.5713	0.6531	0.3165	0.5805	**0.7460**		5	0.7383	0.6552	0.6545	0.3236	0.6861	**0.7401**
	8	0.7762	0.7027	0.3881	0.6404	**0.8299**		8	0.8261	0.7685	0.6602	0.3800	0.5862	**0.8271**
189029	3	0.5597	0.5780	0.4309	0.5800	**0.5960**	Goldpanfire	3	0.5854	0.4379	0.5141	0.4457	0.4822	**0.5854**
	5	0.6296	0.6287	0.3690	0.5780	**0.7843**		5	0.6812	0.5895	0.5797	0.1967	0.6043	**0.6816**
	8	0.6376	0.6231	0.3538	0.6611	**0.8503**		8	0.7868	0.6912	0.5477	0.2182	0.5729	**0.7878**
228076	3	0.5920	0.6862	0.4031	0.6809	**0.7069**	Nyamuragira	3	0.3012	0.2572	0.2680	0.2260	0.2577	**0.3061**
	5	0.7504	0.7649	0.3926	0.7489	**0.8191**		5	0.4327	0.2927	0.3027	0.1887	0.2752	**0.4303**
	8	0.7591	0.7719	0.4261	0.7203	**0.8745**		8	0.5609	0.4428	0.3173	0.1919	0.3117	**0.5704**
247003	3	0.4492	0.4861	0.3002	0.4391	**0.5276**	Rockhouse	3	0.4129	0.3677	0.3603	0.2514	0.3974	**0.4288**
	5	0.5169	0.5809	0.3198	0.5189	**0.6619**		5	0.6712	0.5084	0.5378	0.2539	0.5579	**0.6672**
	8	0.6510	0.5854	0.3208	0.5441	**0.7809**		8	0.7410	0.5604	0.4876	0.1435	0.3655	**0.7410**
326085	3	0.3845	0.5236	0.3927	0.5175	**0.5861**	Yarrabin	3	0.3380	0.2817	0.3133	0.2329	0.3435	**0.3380**
	5	0.5887	0.5844	0.3453	0.5916	**0.7029**		5	0.5503	0.2760	0.3188	0.1383	0.3015	**0.5526**
	8	0.6746	0.6328	0.0873	0.5536	**0.7957**		8	0.6819	0.4737	0.4250	0.1453	0.4233	**0.6851**

Significant values are in [bold].

**Table 8:** Comparison of EPI values across different algorithms on BSDS500 and NASA dataset images.

BSDS500							NASA							
Image name	nT	AO	EO	PSO	WOA	Proposed	Image name	nT	AHA	AO	EO	PSO	WOA	Proposed
120093	3	0.7146	0.7391	0.4135	0.7255	**0.7497**	Kizimen	3	0.5611	0.4859	0.5575	0.2563	0.5250	**0.5613**
	5	0.7657	0.7493	0.3500	0.7423	**0.8335**		5	0.6949	0.5609	0.6408	0.2952	0.5340	**0.6929**
	8	0.8596	0.7835	0.4353	0.7598	**0.8858**		8	0.7697	0.6993	0.5933	0.2913	0.5322	**0.7727**
159002	3	0.4799	0.5872	0.3401	0.5961	**0.6310**	Yellowstone	3	0.6304	0.4680	0.5838	0.2938	0.5868	**0.6287**
	5	0.5713	0.6531	0.3165	0.5805	**0.7460**		5	0.7383	0.6552	0.6545	0.3236	0.6861	**0.7401**
	8	0.7762	0.7027	0.3881	0.6404	**0.8299**		8	0.8261	0.7685	0.6602	0.3800	0.5862	**0.8271**
189029	3	0.5597	0.5780	0.4309	0.5800	**0.5960**	Goldpanfire	3	0.5854	0.4379	0.5141	0.4457	0.4822	**0.5854**
	5	0.6296	0.6287	0.3690	0.5780	**0.7843**		5	0.6812	0.5895	0.5797	0.1967	0.6043	**0.6816**
	8	0.6376	0.6231	0.3538	0.6611	**0.8503**		8	0.7868	0.6912	0.5477	0.2182	0.5729	**0.7878**
228076	3	0.5920	0.6862	0.4031	0.6809	**0.7069**	Nyamuragira	3	0.3012	0.2572	0.2680	0.2260	0.2577	**0.3061**
	5	0.7504	0.7649	0.3926	0.7489	**0.8191**		5	0.4327	0.2927	0.3027	0.1887	0.2752	**0.4303**
	8	0.7591	0.7719	0.4261	0.7203	**0.8745**		8	0.5609	0.4428	0.3173	0.1919	0.3117	**0.5704**
247003	3	0.4492	0.4861	0.3002	0.4391	**0.5276**	Rockhouse	3	0.4129	0.3677	0.3603	0.2514	0.3974	**0.4288**
	5	0.5169	0.5809	0.3198	0.5189	**0.6619**		5	0.6712	0.5084	0.5378	0.2539	0.5579	**0.6672**
	8	0.6510	0.5854	0.3208	0.5441	**0.7809**		8	0.7410	0.5604	0.4876	0.1435	0.3655	**0.7410**
326085	3	0.3845	0.5236	0.3927	0.5175	**0.5861**	Yarrabin	3	0.3380	0.2817	0.3133	0.2329	0.3435	**0.3380**
	5	0.5887	0.5844	0.3453	0.5916	**0.7029**		5	0.5503	0.2760	0.3188	0.1383	0.3015	**0.5526**
	8	0.6746	0.6328	0.0873	0.5536	**0.7957**		8	0.6819	0.4737	0.4250	0.1453	0.4233	**0.6851**

Significant values are in [bold].

**Fig. 10:**
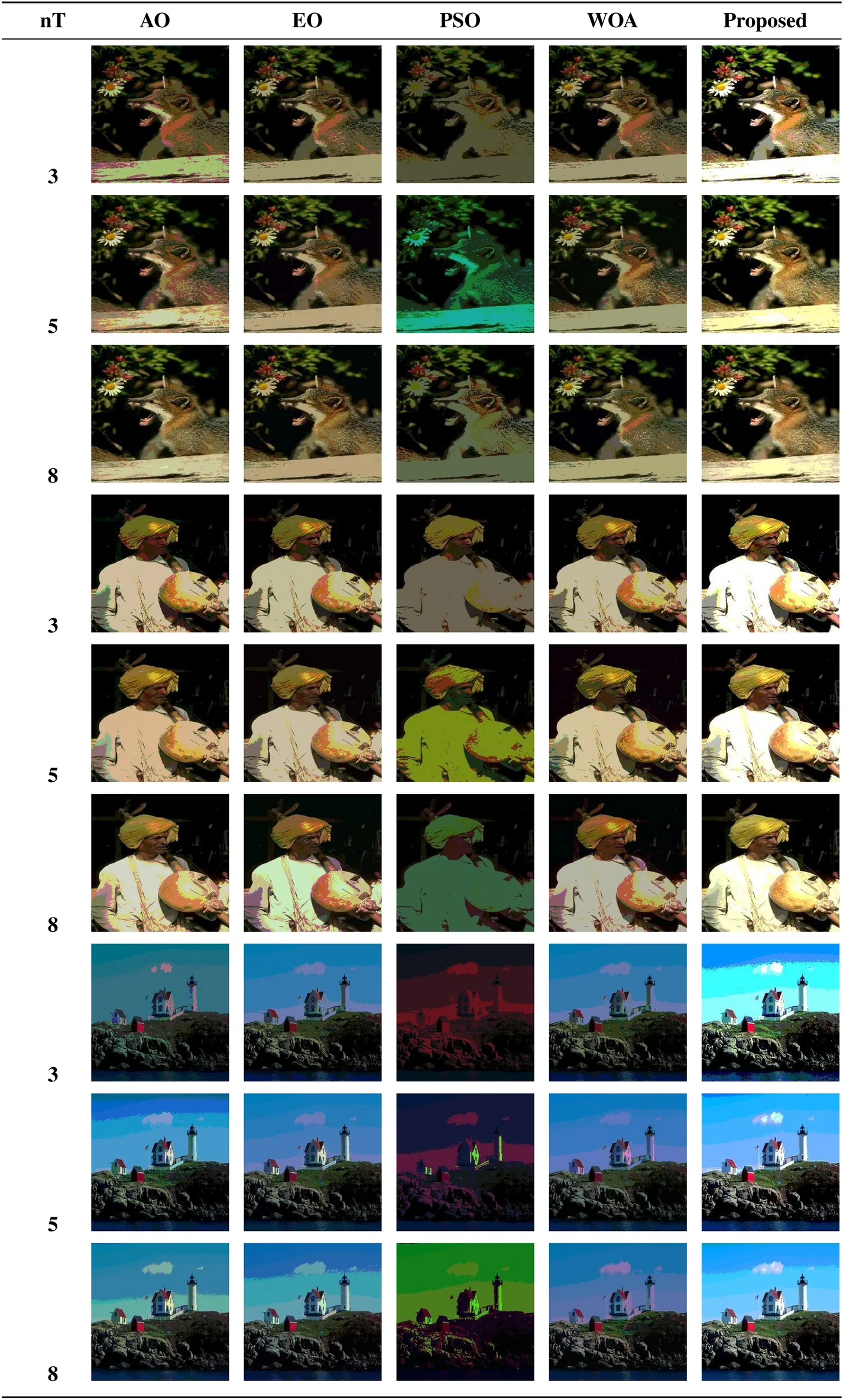
Comparison of AO, EO, PSO, WOA, and Proposed method on BSDS500 228076 image across different threshold levels.

**Table 9:** Comparison of MIF values across different algorithms on BSDS500 and NASA dataset images.

BSDS500							NASA							
Image name	nT	AO	EO	PSO	WOA	Proposed	Image name	nT	AHA	AO	EO	PSO	WOA	Proposed
120093	3	1.8912	1.7055	0.9599	1.5816	**1.7488**	Kizimen	3	1.7463	1.6469	1.7548	0.9402	1.6925	**1.7646**
	5	2.0890	2.0420	1.0707	1.9683	**2.1678**		5	2.2856	2.1603	2.0813	0.9188	1.8907	**2.2863**
	8	2.6051	2.0985	1.4872	1.9912	**2.7443**		8	2.7720	2.6066	2.1059	0.8996	1.9315	**2.7760**
159002	3	1.9146	1.9215	1.4943	1.9171	**1.9215**	Yellowstone	3	1.6799	1.7077	1.7911	1.2009	1.7054	**1.7911**
	5	2.3297	2.2735	1.6924	2.2526	**2.3341**		5	2.1531	2.1349	2.1259	1.2571	**2.1796**	2.1525
	8	2.6376	2.3404	1.7345	2.4022	**2.8271**		8	2.6771	2.4281	2.3250	1.2030	2.0639	**2.6866**
189029	3	1.8287	1.8320	1.4179	1.8179	**1.8320**	Goldpanfire	3	1.7040	1.6760	**1.8162**	1.6640	1.8145	1.7040
	5	2.1066	2.0949	1.4101	2.0635	**2.2006**		5	2.2478	2.0006	1.9998	0.6271	1.9943	**2.2512**
	8	2.3980	2.3430	1.4503	2.1240	**2.6622**		8	2.7131	2.5107	2.2292	0.8097	2.0769	**2.7915**
228076	3	1.6770	1.8212	1.0939	1.8239	**1.8239**	Nyamuragira	3	1.6792	1.7499	1.6758	1.2350	1.4861	**1.8679**
	5	2.1011	2.1186	1.1369	2.1081	**2.1528**		5	2.3745	2.2249	1.9383	0.7295	1.7529	**2.3745**
	8	2.4548	2.3732	1.1713	2.2461	**2.6979**		8	2.9614	2.8218	2.0984	0.9211	1.9502	**2.9614**
247003	3	1.8257	1.7575	1.3866	1.5812	**1.8038**	Rockhouse	3	1.6909	1.6440	1.7079	0.9525	1.6658	**1.7195**
	5	2.3294	2.1107	1.5963	1.9908	**2.2906**		5	2.2317	2.0277	1.9454	0.9627	1.9469	**2.2317**
	8	2.7367	2.2003	1.5073	2.1092	**2.8566**		8	2.7707	2.7012	1.9619	0.4157	1.6549	**2.8038**
326085	3	1.8286	1.8872	1.4181	1.8844	**1.9441**	Yarrabin	3	1.5929	1.7140	**1.7702**	1.1308	1.6830	1.5929
	5	2.3142	2.3073	1.5370	2.2796	**2.4912**		5	2.0625	2.1795	2.0583	0.4966	1.8071	**2.2103**
	8	2.7744	2.3782	0.7156	2.4622	**3.0246**		8	2.7522	2.5400	2.1856	0.7233	2.1688	**2.7622**

Significant values are in [bold].

**Table 10:** Performance comparison of PSNR/SSIM/FSIM/QILV/CORR/EPI/MIF across different optimization techniques for BSDS dataset.

	AO	EO	PSO	WOA	Proposed
AO	_	0.8/0.8/1/0.8/0.9/0.8/1	1/1/1/1/1/1/1	0.9/0.9/1/1/1/0.9/1	0.0/0/0/0.2/0/0/0
EO	0.3/0.2/0/0.167/0.1/0.2/0	_	1/1/1/1/1/1/1	0.8/0.9/0.8/0.8/0.8/0.8/0.8	0.0/0/0/0/0/0/0
PSO	0.0/0/0/0/0/0/0	0/0/0/0/0/0/0	_	0.083/0/0/0/0/0/0	0/0/0/0/0/0/0
WOA	0.1/0.08/0/0/0/0.1/0	0.16/0.08/0.2/0.2/0.2/0.2/0.2	0.9/1/1/1/1/1/1	_	0/0/0/0/0/0/0
Proposed	1/1/1/0.8/1/1/1	1/1/1/1/1/1/1	1/1/1/1/1/1/1	1/1/1/1/1/1/1	_

**Table 11:** Performance comparison of PSNR/SSIM/FSIM/QILV/CORR/EPI/MIF across different optimization techniques for NASA dataset.

	AO	EO	PSO	WOA	AHA	Proposed
AO	–	0.5/0.8/0.7/0.8/ 0.6/0.4/0.9	0.1/1/1/1/1/1/1	0.6/0.8/0.8/0.8/0.7 /0.4/0.8	0.1/0.3/0/0.4/0/0/0.3	0.1/0.3/0/0.4/0/0/0.3
EO	0.5/0.3/0.333/0.25 /0.4/0.6/0.1	–	0.2/1/1/1/1/0.9/1	0.5/0.7/0.7/0.7/0.7 /0.5/0.75	0.2/0.1/0.1/0.2/0/0/0	0.1/0.1/0.1/0.2/0/0/0
PSO	0.9/0/0/0/0/0/0	0.8/0/0/0/0/0.1/0	–	0.7/0/0/0.2/0/0/0	0.6/0/0/0/0/0/0	0.6/0/0/0/0/0/0
WOA	0.4/0.3/0.2/0.2/0.3 /0.6/0.2	0.5/0.3/0.3/0.3/ 0.3/0.5/0.3	0.3/1/1/0.8/1/1/1	–	0.3/0.1/0.1/0.1/0/0/0.1	0.3/0.1/0.1/0.2/0/0/0.1
AHA	0.9/0.7/1/0.6/1/1/0.8	0.8/0.9/0.9/0.8/1/1/1	0.4/1/1/1/1/1/1	0.8/0.9/0.9/0.9/1/1/0.9	–	0.2/0.5/0.3/0.4/0.3 /0.6/0.3
Proposed	0.9/0.7/1/0.6/1/1/0.8	0.9/0.9/0.9/0.8/1/1/1	0.4/1/1/1/1/1/1	0.8/0.9/0.9/0.8/1/1/0.9	0.8/0.5/0.7/0.6/0.7/0.4/0.7	–

#### Correlation coefficient

The correlation coefficient was used to examine how closely the intensity pattern of the segmented image follows that of the original image. In threshold-based segmentation, a higher correlation value suggests that the overall intensity distribution and image structure are preserved with less distortion. Table 7 reports the correlation coefficient values for the BSDS500 and NASA images. The proposed method shows consistent correlation values across different threshold levels, indicating that the segmented outputs remain closely related to the original images. This behaviour is useful for both natural and satellite images, where maintaining the overall intensity pattern is important for reliable segmentation.

#### Edge preservation index

Edge preservation is an important requirement in multilevel segmentation, as poorly selected thresholds may blur or break object boundaries. EPI was therefore used to compare the edge information retained in the segmented image with that of the original image. A higher EPI value indicates better preservation of important boundary and structural details. The EPI values in Table 8 show that the proposed method preserves edge information more reliably across the tested images. The improvement is more noticeable at higher threshold levels, where some baseline algorithms lose boundary clarity. This result is important for both natural images and satellite images, since clear edges help maintain object separation and spatial details after segmentation.

**Table 12:** Performance comparison of multilevel RGB threshold values on BSDS500 dataset using various optimization techniques.

Image name	nT	AO			EO			PSO			WOA			Proposed method		
		R	G	B	R	G	B	R	G	B	R	G	B	R	G	B
120093	3	69 125 189	58 118 194	33 70 120	63 103 181	33 93 163	32 77 141	63 103 181	33 93 162	31 76 141	20 77 159	33 92 162	35 77 141	66 119 179	64 116 174	46 92 146
	5	46 46 86 96 178	53 107 116 159 200	23 32 81 120 160	1 22 65 104 180	2 27 58 97 165	10 39 68 114 149	23 62 96 142 196	29 63 97 140 198	30 71 110 143 181	11 41 60 110 195	1 20 63 96 157	3 32 63 86 146	37 72 110 152 197	36 71 106 146 192	30 60 93 132 175
	8	28 31 60 81 85 119 158 209	26 55 59 104 124 159 187 187	9 30 71 109 132 137 138 246	1 1 4 24 63 102 172 255	1 17 31 59 94 145 218 255	1 2 10 35 71 114 150 255	38 55 71 103 113 136 159 187	57 90 120 127 153 188 220 255	10 40 81 102 115 143 171 196	1 1 1 2 8 65 108 187	1 1 1 1 1 38 94 161	1 1 2 3 22 68 111 151	26 51 75 99 126 150 182 212	28 52 74 99 126 152 181 212	21 41 62 85 107 135 166 205
159002	3	28 72 124	22 100 154	39 99 249	27 85 164	27 80 156	22 57 115	27 85 164	27 79 154	22 57 115	29 82 164	27 78 154	21 59 116	48 102 167	50 100 161	30 73 134
	5	14 40 86 126 223	24 70 108 144 157	26 57 101 159 162	7 26 67 113 178	7 27 51 92 150	5 20 43 75 123	26 65 106 160 211	22 55 92 138 187	19 43 75 111 152	15 22 76 118 180	2 5 32 85 158	2 20 45 82 127	30 62 99 140 191	34 66 100 138 187	21 48 81 125 183
	8	15 21 52 77 106 136 162 213	16 35 53 89 97 159 187 216	11 14 34 51 93 125 229 240	1 1 5 20 30 63 120 178	1 1 1 10 26 72 113 182	1 1 1 5 18 40 71 122	24 60 79 105 122 129 184 184	6 26 60 100 151 164 204 255	19 43 76 106 142 177 241 255	1 1 1 25 39 54 99 176	1 1 1 1 2 33 74 159	22 58 115 240 247 253 255 255	21 42 64 87 114 143 179 211	22 40 63 87 108 136 171 209	15 28 49 71 95 125 155 196
189029	3	17 88 183	31 108 190	29 79 189	29 96 191	27 90 185	28 80 149	32 99 192	27 90 185	28 80 149	32 99 191	34 97 186	27 80 152	51 112 181	48 105 174	37 87 151
	5	18 52 136 208 229	18 66 123 169 181	27 61 126 146 193	6 25 71 125 199	22 47 81 136 208	4 23 55 103 162	21 57 103 160 218	21 46 90 147 209	20 42 74 117 169	28 51 114 156 220	27 84 146 194 208	25 56 71 116 179	32 67 109 154 205	32 66 107 151 200	25 54 91 134 183
	8	17 39 39 46 57 115 163 232	24 53 64 109 144 164 205 250	28 42 57 59 70 105 132 166	1 1 4 29 61 110 187 209	1 1 4 23 41 79 165 207	5 22 42 68 109 164 228 242	25 42 61 101 126 128 163 199	16 25 39 66 85 106 142 180	20 31 50 96 108 155 186 214	6 20 49 100 171 232 232 232	2 22 49 67 110 153 163 215	1 1 5 21 57 82 109 167	20 38 65 94 125 157 190 225	22 42 65 92 122 154 186 218	16 35 57 82 114 141 176 209
228076	3	29 76 128	24 54 112	37 75 150	18 56 115	21 61 121	29 80 170	25 74 212	21 61 121	29 80 170	18 55 105	5 32 102	27 79 170	35 82 151	39 86 150	39 86 156
	5	25 68 94 126 217	29 83 143 157 214	5 21 62 125 192	6 20 44 75 141	1 24 63 120 222	4 25 57 105 184	15 42 75 119 212	21 52 91 132 163	16 36 65 109 189	20 53 73 119 187	24 49 87 129 230	11 31 50 106 190	22 48 81 121 177	27 56 91 134 190	24 50 81 123 179
	8	8 20 45 62 109 133 180 230	19 57 66 128 161 197 239 246	5 25 45 56 76 93 101 146	1 4 17 32 57 94 186 255	1 1 8 26 58 107 150 222	1 4 20 47 76 104 185 255	5 25 48 70 95 132 212 235	20 51 79 117 151 169 219 224	20 42 73 96 127 170 212 235	1 1 1 3 27 48 91 133	1 1 1 2 3 27 62 119	6 16 60 102 185 207 255 255	14 28 43 61 81 112 151 191	18 35 51 71 93 120 156 203	15 29 49 69 92 125 161 197
247003	3	62 109 188	41 101 159	30 55 109	22 69 136	36 94 154	44 76 126	22 69 137	36 93 153	44 76 126	26 69 135	38 91 153	43 75 125	54 99 163	56 105 163	50 91 150
	5	38 67 77 132 168	59 73 96 146 218	44 69 80 136 252	4 21 57 90 148	1 22 76 109 158	14 37 56 88 132	28 63 86 115 160	19 56 87 122 174	31 57 89 136 251	6 26 58 107 170	6 50 94 131 198	10 26 53 96 143	33 63 95 138 190	36 72 103 140 189	31 56 87 126 179
	8	26 55 80 107 144 166 169 235	37 59 94 120 143 147 187 253	30 65 71 91 129 185 215 218	1 1 5 12 24 62 99 154	1 4 31 61 95 149 255 255	1 1 2 22 48 63 98 137	33 62 80 102 132 136 179 224	10 17 25 71 106 138 185 248	17 43 64 90 123 166 171 212	22 50 117 187 187 187 187 187	12 33 87 133 238 255 255 255	7 18 31 51 93 107 107 122	22 42 61 80 101 130 166 207	23 49 70 92 114 142 176 210	23 43 62 80 102 128 160 201
326085	3	70 103 169	50 88 178	37 64 146	39 103 176	29 88 167	27 71 149	39 102 175	29 88 166	27 71 149	43 97 168	31 88 162	27 71 149	57 110 171	50 98 162	41 83 151
	5	37 79 120 170 204	27 38 98 135 178	13 37 67 188 203	15 30 75 124 174	2 26 71 111 179	14 38 63 90 162	26 67 106 157 203	23 60 91 125 187	22 53 83 112 173	24 43 85 139 186	32 52 93 121 195	19 49 80 120 157	38 73 107 145 194	31 64 95 131 185	25 52 82 121 178
	8	30 49 62 104 108 142 185 237	15 33 62 100 143 146 197 234	7 39 70 92 108 135 152 172	1 17 20 22 41 83 125 174	1 3 16 33 64 102 129 177	1 1 4 17 46 71 100 164	20 32 45 90 137 156 176 199	23 53 72 90 109 128 151 187	16 43 74 100 131 169 174 185	1 18 52 96 160 205 205 205	1 1 10 28 53 93 138 203	1 10 18 58 91 95 125 178	23 46 69 90 118 142 173 210	21 43 63 81 104 129 158 204	19 33 50 67 90 117 156 201

**Table 13:** Performance comparison of multilevel RGB threshold values on NASA dataset using various optimization techniques.

Image name	nT	AHA			AO			EO			PSO			WOA			Proposed method		
		R	G	B	R	G	B	R	G	B	R	G	B	R	G	B	R	G	B
Kizimen	3	77 142 194	55 100 150	40 83 134	72 141 210	56 102 165	41 62 95	71 153 203	53 99 148	27 73 122	69 141 196	54 100 150	33 76 124	69 132 199	51 87 145	28 69 122	77 142 194	55 100 150	40 83 134
	5	52 97 141 177 209	39 70 103 139 174	26 53 85 120 162	17 78 144 183 196	64 96 129 172 213	12 54 76 94 130	2 74 141 196 251	12 55 88 117 161	16 42 74 106 140	52 73 95 109 176	21 66 92 126 158	22 59 99 138 231	69 86 107 173 191	2 28 82 103 150	2 2 35 77 128	52 99 143 179 210	39 70 103 140 175	26 52 84 118 162
	8	26 58 91 122 151 176 199 222	25 47 68 91 115 140 164 194	17 35 54 76 100 124 150 189	43 53 81 119 133 173 209 240	21 49 63 91 115 144 157 245	18 50 54 97 120 134 148 176	1 1 1 2 11 69 141 184	1 7 9 47 94 115 136 171	3 8 16 17 39 68 96 133	54 62 74 83 91 92 109 184	20 21 22 51 75 121 159 246	28 47 67 69 76 126 225 237	1 2 9 13 21 73 115 197	27 39 51 95 147 184 255 255	2 3 24 56 83 134 134 134	23 53 83 113 149 175 198 219	28 48 69 92 119 141 163 194	20 39 59 81 103 126 153 200
Yellowstone	3	28 78 143	64 117 155	47 78 140	15 38 148	46 113 141	15 48 74	11 55 136	52 114 148	28 49 77	14 61 141	71 117 149	44 68 94	19 68 138	62 121 144	5 47 76	28 78 143	65 117 155	47 78 140
	5	16 43 78 121 171	45 89 121 149 186	31 51 72 98 154	15 65 120 151 181	59 117 153 172 204	34 39 57 82 179	6 30 56 102 166	2 35 96 127 155	11 41 63 90 250	6 18 51 117 158	14 35 91 120 151	13 44 66 92 202	6 22 57 131 189	29 78 106 143 171	12 22 45 73 97	16 44 81 124 175	45 89 121 150 187	31 50 72 99 153
	8	10 25 44 67 93 122 155 192	27 59 88 110 128 147 167 201	21 37 51 66 82 101 134 188	8 39 77 83 119 136 195 212	3 50 55 81 119 147 170 176	27 42 59 90 104 143 149 205	1 2 5 18 45 109 165 255	1 1 6 41 58 111 138 159	1 7 17 39 65 89 176 232	10 42 67 81 84 122 149 176	56 77 110 116 125 139 144 182	17 46 71 100 142 189 195 217	5 17 17 63 133 162 255 255	5 16 32 117 146 240 255 255	1 1 2 5 17 41 63 90	9 22 40 59 89 118 154 189	27 59 89 105 123 142 160 196	24 38 53 68 84 105 138 195
Goldpanfire	3	53 103 158	74 127 172	57 110 149	55 89 166	101 144 209	90 109 166	42 95 157	85 136 182	76 106 132	46 99 160	87 132 176	96 119 145	65 115 160	29 108 165	106 119 136	53 103 158	74 127 172	57 110 149
	5	34 67 101 138 180	46 89 124 156 189	47 91 115 139 182	33 68 123 166 228	17 80 123 150 169	29 45 55 102 134	7 23 70 102 161	2 35 95 143 183	1 17 94 118 142	9 27 60 115 175	60 94 119 142 181	47 96 115 136 182	22 76 132 182 255	50 91 128 177 177	12 38 95 118 141	34 67 102 138 181	47 89 126 157 189	45 91 115 139 182
	8	23 45 67 90 114 140 167 199	29 61 89 113 135 157 179 204	28 61 86 103 119 135 155 194	12 28 60 88 113 154 221 234	39 77 96 121 131 161 168 181	2 29 69 83 106 130 154 187	6 8 19 32 75 97 118 170	1 1 2 34 104 120 147 185	1 2 6 14 36 95 118 144	6 35 68 91 141 144 166 204	54 66 81 89 97 133 168 255	34 58 107 139 201 204 212 226	36 61 108 123 170 255 255 255	1 1 7 17 83 115 138 198	1 23 30 35 38 90 115 146	23 47 66 87 111 136 168 202	29 61 89 117 136 162 181 205	27 58 87 102 121 137 158 196
Nyamuragira	3	65 116 166	58 121 178	59 108 166	28 115 145	19 138 187	44 91 156	9 88 150	10 122 180	10 78 146	14 88 148	23 123 179	12 78 146	1 19 119	34 118 181	21 78 148	65 116 166	58 121 178	59 108 166
	5	35 76 112 147 185	34 77 116 151 194	30 66 102 141 189	28 58 105 136 150	10 115 136 167 219	15 27 58 102 180	16 66 101 156 255	3 29 115 173 255	12 16 64 107 161	20 49 79 119 184	33 57 102 138 191	13 61 101 133 181	1 2 63 83 154	3 15 60 122 189	1 1 15 81 143	35 76 112 147 185	34 77 116 151 194	30 66 102 141 189
	8	26 55 80 104 128 152 177 207	22 51 81 107 131 154 182 216	23 48 71 94 117 143 173 210	9 54 77 96 120 145 186 235	2 31 92 121 147 165 196 208	6 38 55 104 124 166 192 235	1 2 11 75 116 162 254 255	1 1 1 1 3 114 135 182	1 1 5 17 63 101 149 255	34 43 82 92 130 188 189 231	29 36 42 56 59 72 115 163	28 41 45 51 78 137 173 255	3 8 34 82 136 163 250 250	1 1 1 1 2 42 115 182	3 61 110 167 255 255 255 255	26 55 80 104 128 152 177 207	22 51 81 107 131 154 182 216	23 48 71 94 117 143 173 210
Rockhouse	3	66 120 173	66 113 161	65 113 162	104 146 167	99 101 154	81 132 196	85 126 179	67 104 159	62 96 158	90 137 183	75 121 166	69 111 161	3 115 176	44 98 162	79 141 170	66 120 173	66 113 161	65 113 162
	5	39 80 117 154 188	41 76 110 143 178	41 77 111 145 180	68 109 145 177 247	12 66 112 169 247	41 88 127 163 190	25 79 113 152 193	13 25 72 123 167	3 51 85 115 155	26 78 114 154 193	66 92 120 164 234	21 67 107 147 179	2 50 99 135 177	1 18 74 118 164	32 61 106 142 181	39 80 117 154 188	41 76 110 143 178	41 77 111 145 180
	8	24 52 77 101 128 154 178 202	26 52 75 99 123 147 171 199	26 53 77 100 124 148 173 200	21 32 73 108 120 153 178 201	55 63 75 85 117 149 190 230	66 92 111 148 169 183 190 197	1 1 1 2 27 99 147 186	1 1 6 11 27 70 116 166	1 1 4 13 64 99 154 187	22 41 80 85 108 130 181 247	36 39 49 67 98 138 181 241	69 77 116 119 125 156 200 245	7 7 20 30 59 136 136 173	50 83 160 160 160 160 160 160	3 82 136 153 154 154 154 154	24 52 77 101 128 154 178 202	26 52 75 99 123 147 171 199	26 53 77 100 124 148 173 200
Yarrabin	3	38 97 167	56 112 168	51 105 158	12 93 165	67 123 180	81 112 178	17 88 170	53 110 168	93 122 156	21 90 171	64 118 170	89 115 148	35 72 156	76 132 174	23 100 135	38 97 167	56 112 168	51 105 158
	5	22 57 99 146 195	35 74 113 150 187	41 83 108 136 181	27 55 135 167 209	47 92 97 134 181	75 96 119 172 232	1 11 40 113 185	20 42 95 140 180	1 15 87 112 145	14 27 44 110 176	33 84 127 160 195	41 75 94 118 152	1 18 58 136 187	1 2 42 108 166	1 1 13 100 137	22 57 99 146 195	35 74 113 150 187	41 83 108 136 181
	8	12 30 53 80 110 142 176 212	21 46 71 98 125 150 175 203	22 52 78 95 112 131 157 198	8 27 88 141 187 206 226 239	5 26 63 84 120 149 187 254	11 91 111 130 171 205 212 223	1 1 3 17 45 103 157 209	1 2 3 11 43 95 132 172	5 12 28 87 113 147 248 254	9 30 43 46 55 76 115 167	58 81 98 110 112 130 149 194	23 26 31 49 83 107 128 159	1 1 11 32 89 149 203 255	24 34 75 97 111 129 170 255	96 129 183 238 255 255 255 255	12 30 53 80 110 142 176 212	21 46 71 98 125 150 175 203	22 52 78 95 112 131 157 198

**Fig. 11:**
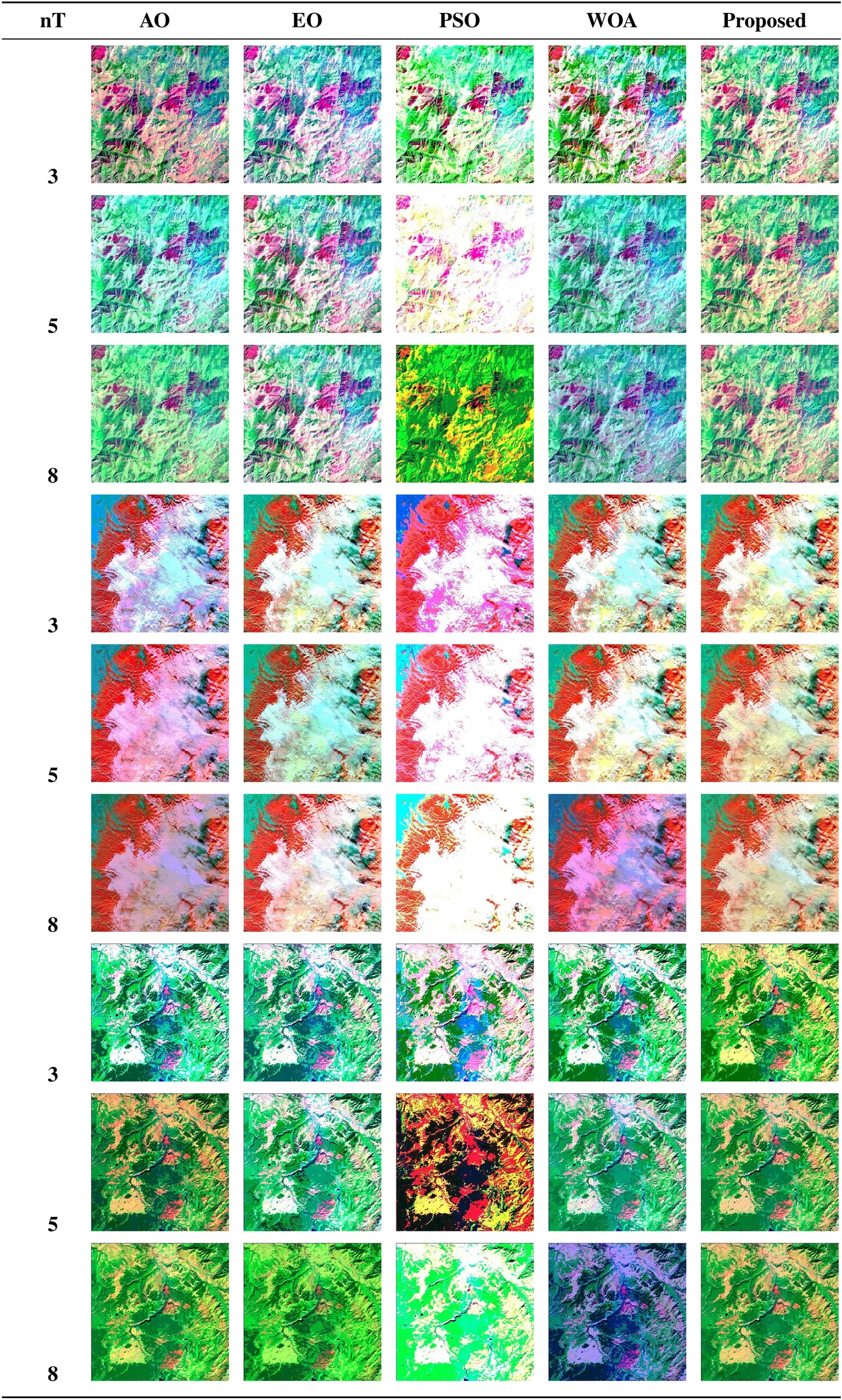
Comparison of AO, EO, PSO, WOA, and Proposed method on NASA image Goldpanfire across different threshold levels.

#### Mutual information factor

MIF was used to analyze the extent of information conservation in the segmented image compared to the original one. For multilevel thresholding, an increase in MIF signifies conservation of information concerning intensity and loss of less information through segmentation. From Table 9, it is evident that the MIF values confirm the validity of the proposed technique. Compared to the baseline techniques, the proposed algorithm better conserves the image information in most cases across the test datasets of BSDS500 and NASA images. This is particularly significant when dealing with complicated images since too much thresholding tends to destroy minute details in the image.

#### Comparison algorithms channel wise threshold distribution

Threshold values obtained for the RGB color components of the BSDS500 and NASA datasets are illustrated in Tables 12 and 13. Thresholds display variations for each color component owing to differing properties such as light intensities, textures, and contents of the images. In comparison with conventional techniques, the threshold sets obtained using the proposed technique are more consistent especially for high threshold values, since the search space is harder in such cases.

### Pairwise statistical comparison of competing algorithms

A pairwise comparison was also carried out using PSNR, SSIM, FSIM, QILV, CORR, EPI, and MIF to give a broader view of segmentation quality. Tables 10 and 11 summarize the results for the BSDS500 and NASA datasets, respectively. Overall, the proposed method shows more consistent performance than the baseline algorithms across most of the evaluated metrics. The results suggest that the method is able to preserve reconstruction quality, structural details, edge information, and useful image content across both natural and satellite images.

### Qualitative evaluation

A qualitative visual comparison was also conducted to further evaluate the segmentation performance besides the quantitative evaluation. The representative results of various images are provided to emphasize clarity of boundaries, consistency of regions and maintenance of fine details. Figure 6 indicates that at an eight level threshold, image 247003 in the BSDS500 dataset, the proposed method exhibits better results in terms of boundary preservation and region refinement than AO, EO, PSO, and WOA. Figure 7 shows how the selected threshold vectors partition the histogram and energy curve of the BSDS500 image into *nT+1* regions, supporting the visual segmentation result. The Yellowstone satellite image in Fig. 8 represents the image at third level threshold, indicating that the proposed method will consistently retain the structural information and minimize the over segmentation. Figure 9 presents the same threshold partitioning for the NASA Goldpanfire image, showing how the energy curve helps maintain stable region separation at the selected threshold level. The comparative outcomes in various threshold levels of BSDS500 and NASA images, in Figs. 10 and 11 respectively, confirm that the proposed approach is always able to draw sharper boundaries and higher homogeneity in comparison to the existing methods. These visual observations also supplement the quantitative findings, supporting the high efficiency and versatility of the proposed segmentation method in natural and satellite images.

### Visual comparison

**Fig. 12:**
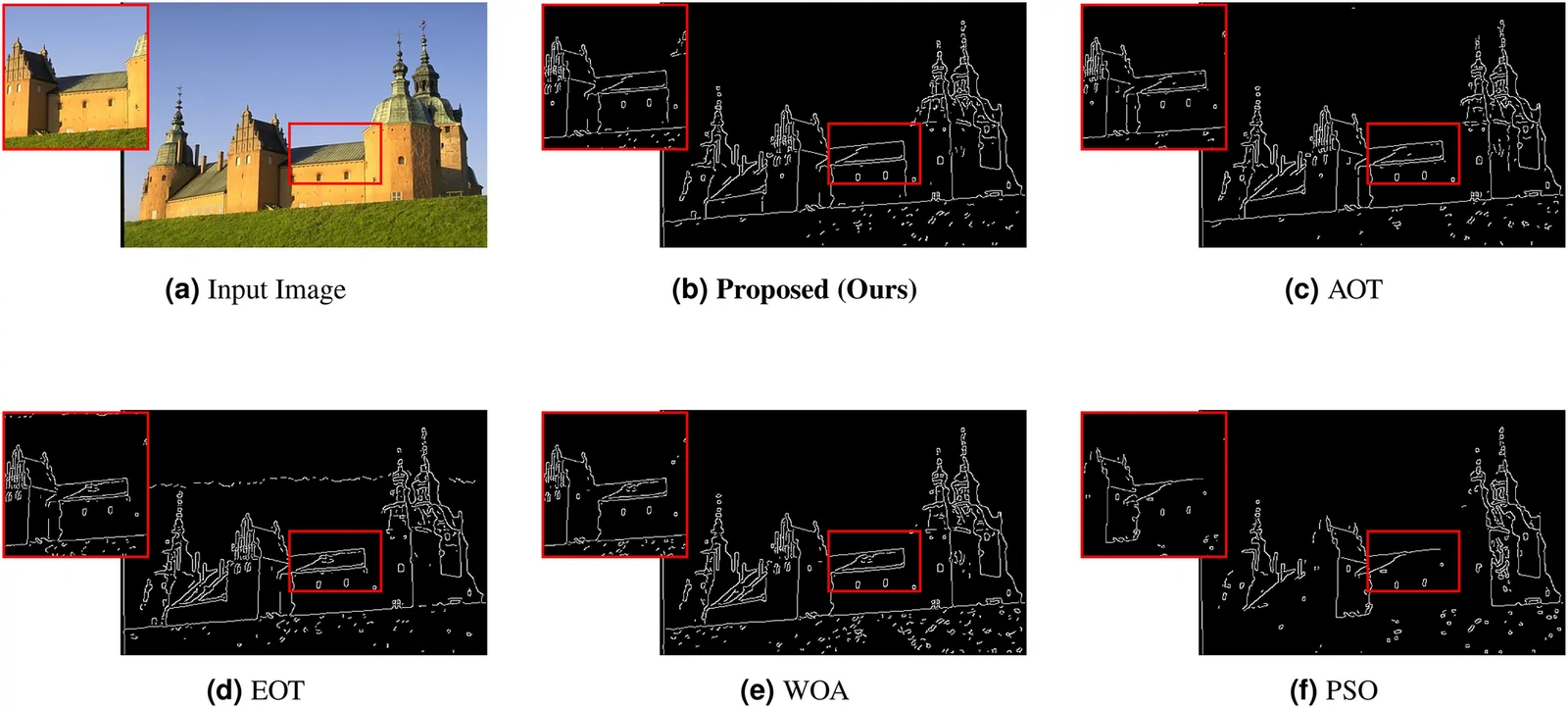
Visual comparison of edge preservation with zoomed rectangular insets. The central region of each image is enlarged inside the rectangular inset to highlight differences in boundary continuity.

In order to evaluate the ability of the proposed technique in retaining edges, magnified images from difficult areas containing complex textures and weak boundaries are shown in the red boxed area in Fig. 12. In these difficult regions, it has been observed that the conventional metaheuristics algorithms, WOA and PSO, cause heavy edge distortion or over-segmentation. The new model, on the other hand, maintains sharp edges throughout. The superiority of the method is highlighted even more when used on architectural images, where the retention of the edge between the building and background is much better than AO and EO approaches.

### Ablation study of initialization strategies

In order to clarify the significance of each mathematical sequence used during the initialization process, an ablation study is conducted. All Latin Hypercube, Sobol, Halton, and Sierpinski algorithms have been tested separately for one complex natural image from the BSDS500 dataset (Image 120093) with the usage of an 8-level thresholding. In order to make results comparable, the same number of iterations and population sizes were used for all methods. The values of convergence time and the resulting fitness are shown in Table 14. It can be concluded that the usage of only one initialization sequence limits the algorithm significantly. For instance, the usage of the Latin Hypercube strategy leads to the shortest convergence time (2.19 seconds), although its fitness value equals to 0.3615 and means the tendency to converge into a local minimum. Moreover, even though both Sobol and Halton sequences require a greater amount of time to execute, they still do not guarantee the highest accuracy of the algorithm. By combining four mathematical sequences together, the suggested algorithm ensures both a reasonable execution time of 3.86 seconds and the highest fitness value of 0.3495.

**Table 14:** Comparison of convergence time and final fitness.

Method	Convergence time (s)	Final fitness
Latin hypercube	2.1964	0.3615
Sobol	5.9784	0.3507
Halton	4.4085	0.3518
Sierpinski	3.8724	0.3552
Proposed	3.8636	0.3495

### Computational time and efficiency analysis

To evaluate the practical efficiency of the compared algorithms, we measured the average time required to segment a single image from the NASA dataset using an 8-level threshold. All experiments were conducted on the same hardware platform to ensure consistency. For a fair comparison focused on the search behaviour of the algorithms, execution time was recorded under the same experimental settings for all methods. The resulting average performance values and runtimes are reported in Table 15.

**Table 15:** Average performance and execution time comparison on the NASA dataset using 8-level thresholding.

Method	PSNR	SSIM	FSIM	QILV	CORR	EPI	MIF	Runtime (s)
AO	33.1760	0.9822	0.8848	0.9283	0.9591	0.5794	2.4373	1.4033
EO	31.4021	0.9659	0.8243	0.8739	0.9369	0.5014	2.0034	0.8179
PSO	33.7533	0.8616	0.5797	0.6466	0.6509	0.2486	0.8475	1.0266
WOA	32.7828	0.9510	0.7856	0.8380	0.9249	0.4658	1.8716	0.7249
Proposed	**37.5360**	**0.9879**	**0.9259**	**0.9466**	**0.9795**	**0.7233**	**2.6157**	1.4578

Significant values are in [bold].

As shown in Table 15, simpler baseline methods such as WOA and EO achieve lower execution times. The proposed AHA-GDA method requires slightly more time, with an average runtime of 1.4578 seconds. This modest increase in computational cost is mainly due to the channel wise energy curve computation, where spatial energy values are calculated separately for the red, green, and blue image channels. Although these components introduce a modest overhead, they play an important role in improving search space exploration and reducing the risk of being trapped in local minima.

At the same time, the proposed method achieves the highest average values for PSNR, SSIM, FSIM, QILV, CORR, EPI, and MIF on the NASA dataset at 8-level thresholding. Therefore, the small increase in runtime is justified by the improvement in final segmentation performance. Overall, the proposed method provides a reasonable trade-off between computational time and segmentation accuracy for complex satellite image segmentation.

### Ablation study

To support the inclusion of these three components, namely structured initialization, GDA refinement, and the energy curve, in the AHA framework, an ablation study was carried out. The ablation results for image 201080 at 8-level thresholding show that the complete model achieves the best performance across all evaluation metrics. Table 16 presents the quantitative results for test image 201080 when these components are omitted in a systematic manner. As observed from Table 16, each of the components plays an individual and unique role in ensuring segmentation accuracy. The exclusion of structured initialization leads to a sharp drop in the quality of signal reconstruction since PSNR drops from 39.0212 to 36.0021. On the other hand, the exclusion of the energy curve component leads to a decrease in the spatial and structural quality as revealed in SSIM of 0.9890 and QILV of 0.9640. The findings show that the energy curve is very critical in establishing context between the neighbors, which cannot be fully captured by the histogram. Finally, omitting GDA leads to lower FSIM value of 0.8779, indicating that GDA helps the solutions escape local optimum traps during later iterations.

**Table 16:** Ablation study results for image 201080.

Method	Image name	SSIM	FSIM	PSNR	QILV	CORR	EPI	MIF
Without initialization	201080	0.9944	0.8819	36.0021	0.9833	0.9872	0.8926	2.6450
Without GDA	201080	0.9936	0.8779	37.3447	0.9818	0.9860	0.8921	2.6546
Without energy curve	201080	0.9890	0.8797	37.5668	0.9640	0.9879	0.8884	2.8258
With Initialization+GDA +Energy Curve	201080	0.9953	0.8838	39.0212	0.9858	0.9885	0.8931	2.9054

### Statistical significance analysis

In order to prove that the benefits in image segmentation provided by the proposed method are not due to the randomness of the metaheuristic technique but are mathematically sound, the non-parametric Friedman test was used. The analysis is carried out on the NASA dataset at the 8-level threshold. The segmentation results for all test images are evaluated using PSNR, SSIM, and FSIM measures. Table 17 demonstrates that, in all cases, the mean ranks indicate that the proposed method performs better than the competing methods. Also, the p-values for all three measures were found to be less than 0.001. Since these p-values are significantly smaller than the alpha value of 0.05, null hypothesis cannot be accepted with greater than *99.9%* confidence.

**Table 17:** Friedman test-based statistical significance analysis.

Metric	Proposed	AO	EO	PSO	WOA	Friedman $\chi ^2$	p-value	Significance
PSNR	1.80	3.23	3.85	2.73	3.40	39.18	*< 0.001*	Highly significant
SSIM	1.28	2.10	3.15	4.95	3.53	126.18	*< 0.001*	Highly significant
FSIM	1.03	2.08	3.20	4.98	3.73	147.56	*< 0.001*	Highly significant

Lower mean rank values indicate superior performance, and the proposed method achieved the best rank across all evaluation metrics.

## Conclusion

This paper has suggested a hybrid multilevel image segmentation model that is effective in integrating the methods of initialization, energy curve, the AHA and the GDA. Latin Hypercube, Sobol, Halton and Sierpinski sequences benefiting the initialization phase provided diversity of the population and improved the exploration of AHA through well dispersed candidate solutions. Also, structural consistency was maintained and the coherence of segmented regions enhanced because of the integration of spatial contextual information. The GDA was used as an adaptive acceptance mechanism, which further optimized the exploitation process by better accepting solutions and ensuring that the search did not get stuck in local minima. MCEM is employed as the objective function to effectively guide the threshold selection process and achieve superior fitness values. The experiments on benchmark color images determined that the suggested algorithm attained higher thresholding accuracy, and improved segmentation as seen on the generated visually consistent segmented images of the state of the art algorithms. Future extensions may include large scale applications such as medical diagnostics, hyperspectral image processing, and real time video segmentation, where energy curve representation could further enhance interpretability and decision making.

## Data Availability

The corresponding author can provide the data validating the study’s conclusions upon reasonable request.
